# Improving the identification of antigenic sites in the H1N1 influenza virus through accounting for the experimental structure in a sparse hierarchical Bayesian model

**DOI:** 10.1111/rssc.12338

**Published:** 2019-02-03

**Authors:** Vinny Davies, William T. Harvey, Richard Reeve, Dirk Husmeier

**Affiliations:** ^1^ University of Glasgow UK

**Keywords:** Antigenic variability, Bayesian hierarchical models, Influenza virus, Latent variable models, Markov chain Monte Carlo sampling, Mixed effects models, Spike‐and‐slab prior, Widely applicable information criterion

## Abstract

Understanding how genetic changes allow emerging virus strains to escape the protection afforded by vaccination is vital for the maintenance of effective vaccines. We use structural and phylogenetic differences between pairs of virus strains to identify important antigenic sites on the surface of the influenza A(H1N1) virus through the prediction of haemagglutination inhibition (HI) titre: pairwise measures of the antigenic similarity of virus strains. We propose a sparse hierarchical Bayesian model that can deal with the pairwise structure and inherent experimental variability in the H1N1 data through the introduction of latent variables. The latent variables represent the underlying HI titre measurement of any given pair of virus strains and help to account for the fact that, for any HI titre measurement between the same pair of virus strains, the difference in the viral sequence remains the same. Through accurately representing the structure of the H1N1 data, the model can select virus sites which are antigenic, while its latent structure achieves the computational efficiency that is required to deal with large virus sequence data, as typically available for the influenza virus. In addition to the latent variable model, we also propose a new method, the block‐integrated widely applicable information criterion biWAIC, for selecting between competing models. We show how this enables us to select the random effects effectively when used with the model proposed and we apply both methods to an A(H1N1) data set.

## 1 Introduction

Human influenza viruses are a major cause of morbidity and mortality world wide, with seasonal epidemics of influenza estimated to result in 3 million–5 million cases of severe illness and 250000–500000 deaths (World Health Organization, 2009). Individuals usually mount an effective antibody‐mediated immune response following infection or vaccination that provides long‐lasting protection against a particular strain of the influenza virus. However, seasonal influenza viruses evolve rapidly and changes to the parts of the virus (termed antigens) that are recognized by the immune system enable the virus population to evade existing immunity and individuals experience recurrent infections. Furthermore the effectiveness of the vaccine, which remains the most effective means of disease prevention, depends on the constituents being well matched to circulating viruses. The continuing antigenic evolution of influenza viruses requires a World Health Organization co‐ordinated global influenza surveillance and response system, which is responsible for the identification of new genetic and antigenic variants among circulating viruses to ensure that influenza vaccine components reflect the antigenic characteristics of circulating viruses (World Health Organization, 2009).

Influenza viruses are classified into three distinct types (A, B and C), of which A and B viruses circulate globally in humans and are responsible for seasonal epidemics. Influenza A viruses are particularly diverse and are further classified into subtypes (e.g. A(H1N1)). The influenza vaccine comprises strains of A(H1N1), A(H3N2) and B viruses predicted to elicit the most effective immune responses against circulating viruses in the forthcoming influenza season (Barr *et al*., 2014). Vaccination provides minimal protection across subtypes and effectiveness within subtype is maximized when the vaccine virus is more antigenically similar to circulating viruses. Genetic mutations cause amino acid substitutions in the surface proteins of the influenza virus that affect recognition by the human immune system. The ever‐changing antigenic characteristics of influenza viruses require that the vaccine formulation is reviewed twice annually and is frequently updated to maintain protection.

The motivation behind this work is to develop models that predict antigenically significant amino acid residues within the influenza surface proteins. An improved understanding of the genetic basis of antigenic evolution has the potential to aid the vaccine selection process in a variety of ways. The development of *in silico* models which can predict both antigenic residues and the likely cross‐protection that is offered by candidate vaccine viruses strains is vital for directing these experiments in an efficient manner and reducing the amount of experimental work that must be carried out. In addition to the identification of emerging antigenic variants, experts must anticipate which viruses are likely to predominate in forthcoming epidemic seasons. Models that improve our knowledge of the contributions of changes to amino acid residues to antigenic evolution have the capacity to enhance the existing evolutionary models that are currently used to predict which strains will increase or decrease in frequency through time (e.g. Łuksza and Lässig (2014)).

To infer the antigenic importance of genetic changes that have occurred during the evolution of the virus we require both genetic data and a measure of antigenic similarity. Antigenic properties of influenza viruses are largely determined by the surface protein haemagglutinin. Human antibodies recognize exposed parts of the haemagglutinin, binding and inhibiting it. Amino acid substitutions (changes) on the surface of the haemagglutinin protein cause loss of recognition by human antibodies, and the haemagglutination inhibition (HI) assay is commonly used for antigenic characterization of circulating viruses (Hirst, 1942; World Health Organization, 2011). The resulting HI titre, which is used as the response in our model, is used to assess the antigenic similarity of a circulating test virus to each of a panel of reference strains that typically include the current vaccine strain and a range of potential future vaccines.

Each HI titre can be associated with genetic data relating to differences between the reference and test viruses that are used in the assay. The contributions of individual amino acid substitutions to antigenic evolution can be predicted by comparing amino acid sequences of the reference and test viruses. In addition to antigenic similarity, HI titres also reflect variation in the binding strength of both antiserum and test virus. Variation in each of these binding strengths can also be modelled by using evolutionary terms. In our model, these terms are used as the explanatory variables and the model also takes into account the structure of these variables, namely that they are the same for any observation that is taken from the same pair of viruses. Additionally the model also takes into account experimental effects that result from the data collection process as random effects.

Various methods have been proposed to account for the experimental variation in the measurements and to select the variables which cause the changes in the measured antigenic variability. Originally Reeve *et al*. (2010) used mixed effects models, e.g. Pinheiro and Bates (2000), to predict the antigenic similarity of foot‐and‐mouth disease virus (FMDV) strains. Reeve *et al*. (2010) first selected the random‐effect components and then added terms to account for the evolutionary history of the viruses. Finally a univariate test for significance was used on the residue variables, with a *p*‐value of less than 0.05 corresponding to an antigenically important residue. A similar method has also been applied by Harvey *et al*. (2016) to influenza A(H1N1), using versions of the data sets that are used here.

Davies *et al*. (2014) then introduced a sparse hierarchical Bayesian model called ‘SABRE’ for detecting relevant antigenic sites in virus evolution and showed how it outperformed the method of Reeve *et al*. (2010). SABRE uses spike‐and‐slab priors, as proposed in Mitchell and Beauchamp (1988), to improve variables selection and to outperform the mixed effects least absolute shrinkage and selection operator the lasso (Tibshirani, 1996; Schelldorfer *et al*., 2011). In SABRE, the spike‐and‐slab priors are integrated into a Bayesian hierarchical mixed effects model, allowing for consistent inference of all parameters and hyperparameters, and inference that borrows strength by the systematic sharing and combination of information; see Gelman *et al*. (2013). Davies *et al*. (2017) improved SABRE through the addition of a biologically significant intercept parameter and increased conjugacy between parameters.

The SABRE models of Davies *et al*. (2014, 2017) do not, however, fully take into account the structure of the data and are not sufficiently computationally efficient to work with the H1N1 data set. The structure of the data comes from the fact that the HI assay is often repeated multiple times for the same reference and test virus pair. Correspondingly, the genetic and evolutionary data will be the same for any two measurements where the same reference and test viruses are used. However, as the full set of explanatory variables explicitly depends on which of the two viruses is used as the reference virus and which was used as the test virus, it is worth noting that a given pair of viruses will give different explanatory variables if the strains that are used as reference and test virus are switched. We can use the described structure to improve the accuracy of SABRE and to increase its computational efficiency such that it can now be used on the H1N1 data set. In the current work we introduce an extended version of SABRE, called the extended SABRE model eSABRE, through the use of a latent variable model which better matches the structure of the data. More precisely we introduce latent variables to represent the underlying HI titre of any given pair of reference and test virus.

In addition to selecting the fixed effects, it is also important to choose the random‐effect components. To do the selection we introduce a variation of the widely applicable information criterion, WAIC (Watanabe, 2010): block integrated WAIC, biWAIC, based on integrated WAIC, iWAIC, as proposed in Li *et al*. (2016). biWAIC takes into account the specific structure of eSABRE and integrates over the latent variables. We describe how this converges to a particular form of cross‐validation (CV) and use a simulation study to quantify the improvement that it offers over non‐integrated WAIC, nWAIC.

In this paper we evaluate the advantages of eSABRE over the previously proposed conjugate SABRE model. We use simulated data sets that mimic the structure of the H1N1 data set to show how it offers an improvement in variable selection, as well as an increase in computational efficiency. We also propose and test biWAIC on the simulated data sets to quantify its improvement in selecting random‐effect components within eSABRE. Finally we apply biWAIC with eSABRE to the H1N1 data set and identify some known and potential antigenic sites, comparing the results with those of Harvey *et al*. (2016).

## 2 Data and previous work

The antigenic data that are analysed comprised pairwise measures of antigenic similarity of viruses of the A(H1N1) subtype obtained by using the HI assay. In these experiments, antiserum that was created by exposing a ferret to a particular reference virus is measured in terms of its ability to inhibit the binding of red blood cells (haemagglutination) by a sample of a second virus: the test virus. The HI assay measures the degree of protection that each reference strain would provide against the test virus by recording the maximum dilution at which antibodies in a sample of antiserum from a ferret that was exposed to a particular reference strain remain able to inhibit a sample of the test virus. A high titre in the test corresponds to a high dilution of the antiserum and therefore a low concentration of the antiserum being sufficient to cause inhibition; a low titre conversely corresponds to a low dilution and a high concentration of the antiserum being required. An antiserum can therefore typically inhibit the virus that is used to produce the antiserum at high dilutions, but lower dilutions (i.e. higher concentrations and hence lower titres) are required to inhibit test viruses that are antigenically more dissimilar. Higher HI titres indicate antigenic similarity, and HI titres typically decrease with increasing genetic distance between the reference and test viruses.

Previously, Davies *et al*. (2017) used the conjugate SABRE method with the following probability distribution to model the HI assay data:

(1) $$y∼N(y|1w_{0}+Dw_{\gamma }+Zb,\sigma_{\varepsilon }^{2}I).$$

In this probability distribution, **y**=(*y*_1_,…,*y*_*N*_)^T^ represents the *N* log(HI) titre measurements. The random‐effects design matrix **Z** is set to be the matrix of factor level indicators with *N* rows and ‖**b**‖ columns, where ‘‖·‖’ indicates the length of the vector and **b** is a column vector of random‐effect coefficients. The explanatory variables **D** are given as a matrix of *J* columns and *N* rows, where *J* is the number of explanatory variables. The explanatory variables contain binary indicators of amino acid changes at different residues or information on the phylogenetic structure. Of the explanatory variables **D**, only the variables which are inferred to be relevant to the prediction of **y**,** D**_***γ***_, are included in distribution (1) dependent on $\gamma ={\left(\gamma_{1},\ldots ,\gamma_{J}\right)}^{T}\in {\left\{0,1\right\}}^{J}$. The relevance of the *j*th column of **D** is determined by $\gamma_{j}\in \left\{0,1\right\}$, where feature *j* is said to be relevant if *γ*_*j*_=1. Similarly **w**_***γ***_ is given as the column vector of regressors, where the inclusion of each parameter is dependent on ***γ***.

However, although the conjugate SABRE method provides a reasonable way of modelling the HI titre, it does not adequately represent the true complexity of the data. Within the data, there are multiple measurements **y** which are taken from the same pair of reference and test viruses, *p*, but they are often carried out under different experimental conditions. In the case of two measurements where the same pair of reference and test virus was used, *y*_1_ and *y*_2_, the experimental conditions, **Z**, for these observations can vary, i.e. **Z**_1_≠**Z**_2_ or **Z**_1_=**Z**_2_. However, the corresponding explanatory variables **D** will remain the same, **D**_1_=**D**_2_, whenever the same pair of reference and test viruses is used. It is this structure that motivates the introduction of the eSABRE method in Section 3.

For each observation *y*_*i*_, the explanatory variables **D**_*i*_ include variables that give the differences in protein structure and evolutionary history between the reference and test viruses. As an individual strain will always have the same protein structure, for any pair of virus strains the differences in protein structure remain identical whenever the experiments are carried out, regardless of which strain is used as the reference strain. More precisely, the explanatory variables **D** that give the differences in the protein structure look at whether there is a presence 1 or absence 0 of an amino acid substitution at each specific residue which is exposed on the surface of haemagglutinin protein. Not all of these amino acid substitutions affect antigenicity but any important changes causing antigenic differences are likely to result in a reduction in the observed HI titre measurements **y**.

The viruses that are studied are descended from an evolutionary process and are therefore not statistically independent entities. Shared evolutionary history means that more closely related viruses tend to share traits and false support for the role of a substitution in antigenic change may arise. To account for shared evolutionary history, the evolutionary or phylogenetic tree representing the relatedness of the viruses studied was incorporated in the analysis by using a phylogenetic comparative method described by Reeve *et al*. (2010). Briefly, for any two viruses in the evolutionary tree, a path can be traced through the tree along the branches that separate them. For each observation *y*_*i*_, evolutionary variables that are associated with each branch of the phylogenetic tree indicate whether a branch does (1) or does not (0) form part of the path separating the reference and test viruses that are used in the HI assay. When we cannot attribute antigenic differences to amino acid changes directly, it may be possible to attribute the variation to one of these evolutionary explanatory variables, representing the point in the evolution of the virus (specifically a branch of the phylogenetic tree) where the antigenic characteristics of the virus changed.

In addition to measuring antigenic similarity, HI titres are affected by the binding strength of both antiserum and test virus. Variation in immunogenicity and avidity result in antisera and viruses respectively that vary in their baseline titres. To account for evolutionary signal in this non‐antigenic variation, which results in systematically higher or lower titres for related viruses, further evolutionary variables that are associated with each branch in the phylogenetic tree indicate whether the branch is present (1) or absent (0) from the evolutionary history of the test and reference viruses. For any given pair of viruses, these variables explicitly depend on which of the two was used to create the antiserum and which was used as test virus (see section 6 of the supplementary materials of Davies *et al*. (2017) for further details).

HI titre measurements usually contain significant experimental variation and it is therefore necessary to include random effects. For the A(H1N1) data set the possible random effects are laboratory conditions, reference virus and test virus. Laboratory conditions account for differences in the experimental conditions that are seen on particular days such as the dilution of reagents. The reference and test virus effects account for antiserum and viruses that tend to have systematically higher or lower HI titres in all assays in which they are used.

### 2.1 Influenza A(H1N1)

Influenza A(H1N1) viruses re‐entered the human population in 1977 and cocirculated with viruses of a second influenza A subtype, A(H3N2), and influenza B viruses until their replacement by a novel, distantly related lineage of A(H1N1) viruses in the 2009 swine origin pandemic (Barr *et al*., 2014). During the period 1977–2009, the influenza vaccine included an A(H1N1) strain which had to be updated on nine occasions to remain antigenically matched to, and therefore capable of protecting the human population from, circulating strains. The data set that is analysed here comprises 43 A(H1N1) viruses collected from 1978 to 2009 that were each used as both as reference strains contributing antiserum to the HI assay and as test viruses. From these viruses, 570 different reference and test virus pairs *p* were tested resulting in 15693 HI titre measurements **y**. The mean standard deviation in log(HI) titre values within each pair of viruses is 0.48 (to two decimal places) and more information about the selection of the virus pairs can be found in section 2 of the on‐line supplementary materials. Once residues with incomplete genetic data had been removed, there were 279 explanatory variables consisting of 53 surface‐exposed residues and 226 variables related to the phylogenetic data.

**Figure 1:**
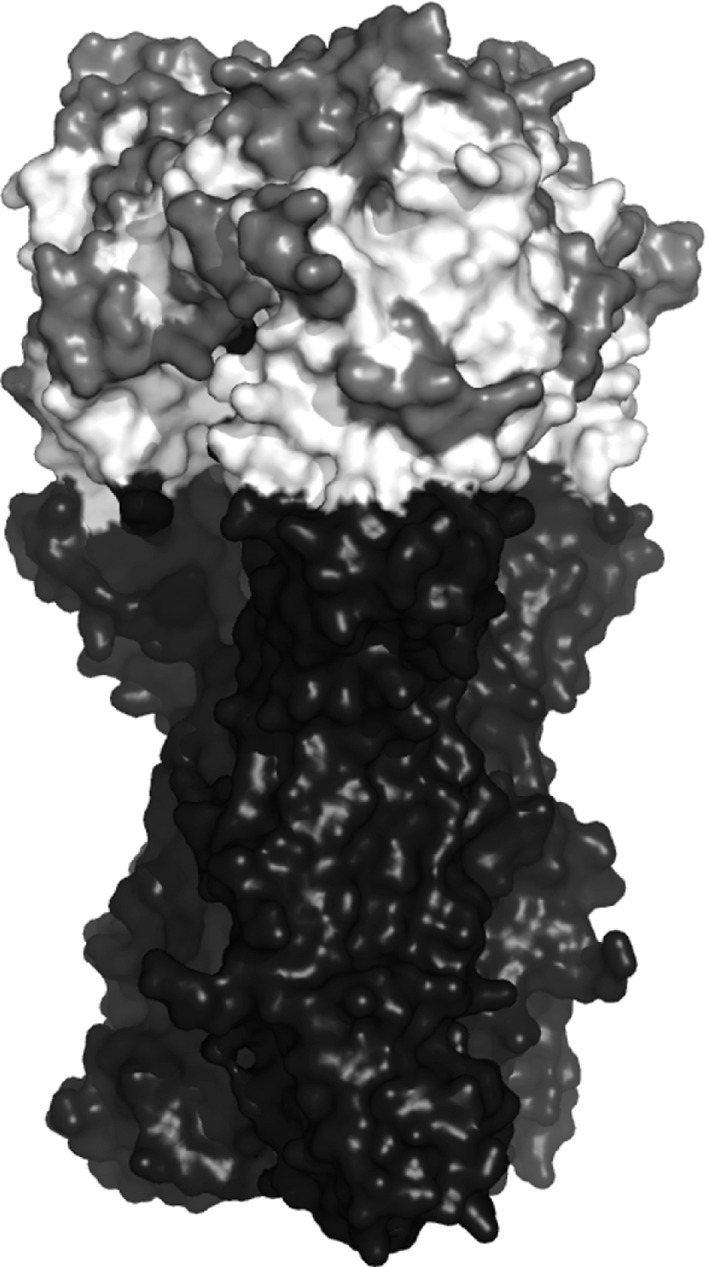
Three‐dimensional structure of the influenza A(H1N1) haemagglutinin protein coloured by antigenic status: haemagglutinin is exposed on the virus surface and is composed of two regions, HA1 and HA2; HA1 is responsible for binding to host cells and is the primary target for the host immune system; known antigenic sites and the receptor binding site where changes are also expected to cause variation in the HI assay are shown in dark grey (proven regions); plausible antigenic regions in the head domain of haemagglutinin are shown in light grey; implausible antigenic regions in the stalk domain are shown in black, as are surface‐exposed areas of the HA2 part of the protein which was not included in our analysis; this model representation of the surface of haemagglutinin is based on the resolved structure of influenza A(H1N1) strain A/Puerto Rico/8/34 (Gamblin *et al*., 2004)

For influenza viruses, the haemagglutinin surface protein is responsible for binding to host cells and is also the major target for neutralizing antibodies (Skehel and Wiley, 2000). Consequently changes to the haemagglutinin structure are usually responsible for the requirement to update vaccine components. The structure of haemagglutinin that is given in Fig. 1 can be broadly divided into the stalk domain which connects to the virus particle and a head domain which contains the residues that are involved in binding to the host cell. Experimental studies have identified that the major antigenic regions of haemagglutinin are protruding areas in the head of the haemagglutinin protein surrounding the receptor binding site (Skehel and Wiley, 2000). For A(H1N1), these experiments have identified four antigenic sites (Caton *et al*., 1982); however, other residues are also known to be important (McDonald *et al*., 2007). We classify residues as proven if they belong to any of the four antigenic sites or have other experimental support for their role in antigenicity (e.g. McDonald *et al*. (2007)), or if they belong to the receptor binding site where substitutions are expected to influence HI titres via changes to virus receptor binding strength. These residues are shown in dark grey in Fig. 1. Other haemagglutinin residues that are exposed on the surface of the head domain are considered to be plausible antigenic residues, whereas residues belonging to the stalk domain are considered unlikely to play a role in antigenic change and are therefore considered implausible. Plausible and implausible antigenic candidate residues are shown in light grey and black respectively in Fig. 1.

## 3 eSABRE

**Figure 2:**
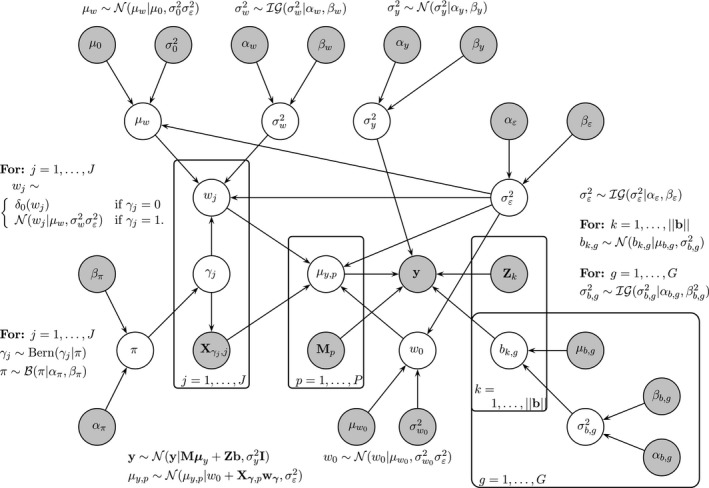
Compact representation of eSABRE as a probabilistic graphical model: 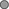, the data and fixed (higher order) hyperparameters: ∘, parameters and hyperparameters that are inferred

eSABRE is based on the conjugate SABRE model that was described in Davies *et al*. (2017) but with a likelihood that better takes into account the data structure. The change in the structure is given in Section 3.1 with the remaining sections defining the prior distributions of eSABRE, keeping to those used for the conjugate SABRE model as closely as possible. Finally, the model is shown as a probabilistic graphical model in Fig. 2 and the parameters are sampled from the posterior distribution by using Markov chain Monte Carlo (MCMC) sampling described in Section 4. The R code for our models is available from https://github.com/vinnydavies/sabre_methods and

https://rss.onlinelibrary.wiley.com/hub/journal/14679876/seriescdatasets

and the data can be obtained from http://researchdata.gla.ac.uk/289/ (Harvey *et al*., 2016).

### 3.1 Latent‐variable‐based likelihood

The probability distribution of the conjugate SABRE method, expression (1), gives a general model which can be used in a variety of contexts; it does not, however, completely account for the structure of the data that are used to model antigenic variability and described in Section 2. Although the experimental conditions, represented by the random effects, usually vary, each pair of reference and test viruses will have the same explanatory variables. As a result we can introduce latent variables ***μ***_**y**_ into the model, where each *μ*_**y**,*p*_ represents the unknown true value of the HI titre of any given pair of reference and test viruses, *p*.

The introduction of the latent variables ***μ***_**y**_ into the model results in the following distribution for **y**:

(2) $$y∼N(y|M\mu_{y}+Zb,\sigma_{y}^{2}I)$$

where **M** is a *N*×*P* design matrix where in each row *i* there is a 1 in the column related to the pair of reference and test strains from which the observation *y*_*i*_ originates, and 0s in the remainder of the row. This ensures that each *y*_*i*_ has the latent variable *μ*_**y**,*p*_ which corresponds to its given pair of reference and test viruses, *p*. The random effects are added to the likelihood as some of these factors, e.g. date, affect measurements at the individual level, i.e. they are different for each *y*_*i*_.

We then wish to infer the values of the HI titre measurements of the pairs of virus strains, ***μ***_**y**_, based on the differences in the protein structure and evolutionary history of the virus:

(3) $$\mu_{y}∼N\left(\mu_{y}|1w_{0}+X_{\gamma }w_{\gamma },\sigma_{\varepsilon }^{2}I\right).$$

In this distribution, **X** is a matrix of explanatory variables for ***μ***_**y**_ and has *J* columns and *P* rows, with **X**_***γ***_ then representing the relevant explanatory variables given ***γ***. ***γ*** and **w**_***γ***_ are defined as in the first paragraph of Section 2. Additionally to this, we also include an intercept parameter $w_{0}$, as we expect high underlying HI titre measurements when the two virus strains that are used are the same, i.e. the explanatory variables are equal to 0. The full model is given graphically in Fig. 2.

The structure that is given by the two main probability distributions of eSABRE, given in distributions (2) and (3), has two major advantages over the main probability distribution of the conjugate SABRE model, given in expression (1). Firstly it allows us to attribute the error to the correct part of the model better. In the HI titre measurements some of the error comes from variability within the experiments, e.g. obtaining multiple different results for the same pair of reference and test viruses under the same experimental conditions, and this is accounted for by $\sigma_{y}^{2}$. Other error will come from the model fit, e.g. our model not truly replicating the underlying biological process, and this is given by $\sigma_{\varepsilon }^{2}$. Improving the attribution of error means that our model matches better with the data collection technique and should lead to more accurate results and an improvement in the identification of antigenic sites.

The second advantage of eSABRE is significantly improved computational efficiency. For example, to run the MCMC simulations to train the model on ‖**y**‖=15693 observations, as discussed in Section 4, it would take SABRE weeks or months to sample the required number of iterations to achieve convergence and a reasonable sample size after burn‐in. In contrast, with the proposed eSABRE model we can achieve these results in a few days. A detailed comparison will be provided in Section 6.3, Table 1. The improvement is a result of reducing the computation that is required to calculate the conditional posterior distribution of ***γ***. In essence, through the introduction of latent variables eSABRE reduces the posterior distribution of ***γ*** to a multivariate Gaussian distribution of dimension ‖***μ***_**y**_‖ (‖***μ***_**y**_‖=570 in the H1N1 data set), as opposed to dimension ‖**y**‖ (‖**y**‖=15693) in SABRE. This is important when there is a large number of variables, *P*=279, and we are required to calculate repeatedly the density of a multivariate Gaussian distribution which scales cubically multiple times for each iteration. This will be discussed in further detail in Section 4.1.

**Table 1:** AUC‐values and CPU times for eSABRE and the conjugate SABRE model applied to the influenza‐inspired simulated data sets†

*Observations*	*Results for eSABRE*			*Results for conjugate SABRE*		
	$\sigma_{y}^{2},\sigma_{\varepsilon }^{2}=0.033$	$\sigma_{y}^{2},\sigma_{\varepsilon }^{2}=0.1$	$\sigma_{y}^{2},\sigma_{\varepsilon }^{2}=0.3$	$\sigma_{y}^{2},\sigma_{\varepsilon }^{2}=0.033$	$\sigma_{y}^{2},\sigma_{\varepsilon }^{2}=0.1$	$\sigma_{y}^{2},\sigma_{\varepsilon }^{2}=0.3$
*(a) AUC‐values*						
500	0.98	0.90	0.82	0.90	0.77	0.64
1000	0.98	0.91	0.82	0.83	0.70	0.59
2000	0.98	0.92	0.83	0.75	0.61	0.58
*(b) CPU times (s) per 1000 iterations*						
500	25	25	47	497	867	444
1000	29	26	36	6931	5623	5546
2000	32	25	43	35231	32243	20904

† The table gives the AUC‐values and the CPU times per 1000 iterations for eSABRE and the conjugate SABRE model. The results come from when the methods were applied to the influenza‐inspired simulated data sets described in Section 6.1.1. with varied numbers of observations. ‡The simulations were run on a cluster with over 30 machines, the majority of which had different specifications. Generally the majority of these machines range from 12 to 40 cores and 8–32 Gbytes of random‐access memory and have a variety of processors.

### 3.2 Noise and intercept priors

The conditional variance of the residuals, given the latent variables, is defined as $\sigma_{y}^{2}$ and represents the variance in the error that is seen in repeated measurements from the HI assay experiments. We give $\sigma_{y}^{2}$ the conjugate prior

(4) $$\sigma_{y}^{2}∼IG\left(\sigma_{y}^{2}|\alpha_{y},\beta_{y}\right)$$

where the hyperparameters *α*_*y*_ and *β*_*y*_ are fixed, as indicated by the grey nodes in Fig. 2.

The variance in distribution (3), $\sigma_{\varepsilon }^{2}$, represents the variance of the discrepancy between the unknown true HI titre values ***μ***_**y**_ and the HI titre estimates ${\hat{\mu }}_{y}$ that are inferred from the fixed effects. In eSABRE we give it the prior

(5) $$\sigma_{\varepsilon }^{2}∼IG\left(\sigma_{\varepsilon }^{2}|\alpha_{\varepsilon },\beta_{\varepsilon }\right)$$

where the hyperparameters *α*_*ɛ*_ and *β*_*ɛ*_ are fixed. $\sigma_{\varepsilon }^{2}$ represents the discrepancy between the unknown true HI titre values for each pair and what is inferred by the fixed effects. $\sigma_{\varepsilon }^{2}$ is also included in the distributions for $w_{0}$, **w**_***γ***_ and $\mu_{w}$ (defined in Section 3.3), making the model conjugate rather than semiconjugate, as discussed in chapter 3 of Gelman *et al*. (2013). The advantage of this information sharing is that the error variance in terms of model fit is reflected in the distribution of the regression coefficients and this has been further explored in Davies *et al*. (2017).

Additionally we also require a prior on our intercept:

(6) $$w_{0}∼N\left(w_{0}|\mu_{w_{0}},\sigma_{w_{0}}^{2}\sigma_{\varepsilon }^{2}\right).$$

We treat the intercept differently from the remaining regressors, wishing to use vague prior settings so as not to penalize this term and effectively to make the model scale invariant (Hastie *et al*., 2009).

### 3.3 Spike‐and‐slab priors

Spike‐and‐slab priors have been used in various contexts and have been shown to outperform *l*_1_‐methods in terms of both variable selection and out‐of‐sample predictive performance (Mohamed *et al*., 2012; Davies *et al*., 2014, 2017). They were originally proposed by Mitchell and Beauchamp (1988) as a mixture of a Gaussian distribution and a Dirac delta spike, but they have also been used as a mixture of two Gaussian distributions (George and McCulloch, 1993, 1997) and as binary mask models, e.g. Jow *et al*. (2014).

The idea behind the spike‐and‐slab prior is that the prior reflects whether the feature is relevant on the basis of the values of ***γ***. In this way we expect that *w*_*j*_=0 if *γ*_*j*_=0, i.e. the feature is irrelevant, and conversely it should be non‐zero if the variable is relevant, *w*_*j*_≠0 if *γ*_*j*_=1. A conjugate Gaussian prior, with $\sigma_{\varepsilon }^{2}$ included for further conjugacy, is then assigned where the feature is relevant and a Dirac delta spike at 0 where it is not:

(7) $$w_{j}∼\left\{\begin{array}{cc} \delta_{0}\left(w_{j}\right) & \text{if}\;\gamma_{j}=0,\\ N(w_{j}|\mu_{w},\sigma_{w}^{2}\sigma_{\varepsilon }^{2}) & \text{if}\;\gamma_{j}=1 \end{array}\right.$$

for *j* ∈ 1,…,*J* and where *δ*_0_ is the delta function. Here we have a spike at 0 and as $\sigma_{w}^{2}\sigma_{\varepsilon }^{2}\to \infty$ the distribution *p*(*w*_*j*_|*γ*_*j*_=1) approaches a uniform distribution: a slab of constant height. The prior for the variance of the parameter is then given by

(8) $$\sigma_{w}^{2}∼IG\left(\sigma_{w}^{2}|\alpha_{w},\beta_{w}\right),$$

where *α*_*w*_ and *β*_*w*_ are fixed; see Fig. 2.

In addition to $\sigma_{w}^{2}$, we use the hyperparameter $\mu_{w}$ to reflect a non‐zero prior mean of the regression coefficients **w**_***γ***_:

(9) $$\mu_{w}∼N\left(\mu_{w}|\mu_{0},\sigma_{0}^{2}\sigma_{\varepsilon }^{2}\right)$$

where the hyperparameters *μ*_0_ and $\sigma_{0}^{2}$ are fixed and $\sigma_{\varepsilon }^{2}$ is again included in the variance for further conjugacy. This specification comes from our biological understanding of the problem. In the H1N1 data set we are likely to observe large HI titre values when the reference and test viruses are the same, represented by the intercept $w_{0}$. Smaller HI titre values will then be seen when the reference and test viruses are different, reflecting the fact that any amino acid changes are likely to reduce the similarity between virus strains and meaning that the regression coefficients *w*_*j*_ are likely to be negative.

The final part of the spike‐and‐slab prior is to set a prior for ***γ***, the hyperparameters which determine the relevance of the variables:

(10) $$p\left(\gamma |\pi \right)=\prod_{j=1}^{J}\text{Bern}\left(\gamma_{j}|\pi \right)$$

where *π* is the probability that the individual variable is relevant. The value of *π* can either be set as a fixed hyperparameter as in Sabatti and James (2005), who argued that it should be determined by underlying knowledge of the problem. Alternatively it can be given a conjugate beta prior

(11) $$\pi ∼B(\pi |\alpha_{\pi },\beta_{\pi })$$

as has been used here. This is a more general model, which subsumes a fixed *π* as a limiting case for *α*_*π*_ *β*_*π*_/{(*α*_*π*_+*β*_*π*_)^2^(*α*_*π*_+*β*_*π*_+1)}→0 and has also been shown to act as a multiplicity correction in Scott and Berger (2010).

### 3.4 Random‐effects priors

In mixed effects models the random effects *b*_*k*,*g*_ are given group‐dependent Gaussian priors. There are *k* ∈ {1,…,*K*} random effects, where we use *g* as a naming convention to say which group the random effect belongs to:

(12) $$b_{k,\;g}∼N\left(b_{k,\;g}|\mu_{b,\;g},\sigma_{b,\;g}^{2}\right).$$

We define this to have a fixed mean *μ*_*b*,*g*_=0 and a common variance parameter $\sigma_{b,\;g}^{2}$, with a conjugate inverse gamma prior for each random‐effects group *g*:

(13) $$\sigma_{b,\;g}^{2}∼IG\left(\sigma_{b,\;g}^{2}|\alpha_{b,\;g},\beta_{b,\;g}\right)$$

where *α*_*b*,*g*_ and *β*_*b*,*g*_ are fixed hyperparameters for each *g* and we define $b∼N(b|0,\Sigma_{b})$ where $\Sigma_{b}=\text{diag}\left(\sigma_{b}^{2}\right)$ with $\sigma_{b}^{2}={\left(\sigma_{b,\;1}^{2},\ldots ,\sigma_{b,\;1}^{2},\sigma_{b,\;2}^{2},\ldots ,\sigma_{b,G}^{2}\right)}^{T}$ such that each $\sigma_{b,\;g}^{2}$ is repeated with length ‖**b**_*g*_‖ as shown in Fig. 2. We are aware that the application of conjugate inverse gamma priors has been disputed by Gelman (2006). However, in our previous work (Davies *et al*., 2017) we found no significant differences in the results from using the more complex prior that is recommended in Gelman (2006).

## 4 Posterior inference

To explore the posterior distribution of the parameters of eSABRE we use an MCMC algorithm. Having chosen conjugate priors where possible means that we can run a Gibbs sampler for the majority of parameters (Ripley, 1979; Geman and Geman, 1984), where we sample the intercept and regression parameters together, and define $w_{\gamma }^{*}=\left(w_{0},w_{\gamma }\right)$, $X_{\gamma }^{*}=\left(1,X_{\gamma }\right)$, **m**=(*μ*_*w*0_,*μ*_*w*_,…,*μ*_*w*_)^T^ and $\Sigma_{w_{\gamma }^{*}}=\text{diag}\left(\sigma_{w^{*}}^{2}\right)$ with $\sigma_{w^{*}}^{2}={\left(\sigma_{w0}^{2},\sigma_{w}^{2},\ldots ,\sigma_{w}^{2}\right)}^{T}$, where each *μ*_*w*_ and $\sigma_{w}^{2}$ is repeated with length $\left\|\gamma \right\|=\Sigma_{j=1}^{J}\;\gamma_{j}$. These conditional distributions are derived in section 1 of the on‐line supplementary materials and given here, where by a slight abuse of notation ***θ*** ^′^ denotes all the other parameters, excluding those on the left of the conditioning bar. The only exception is ***γ***, which is discussed in Section 4.1:

(14) $$\mu_{y}|\theta^{\prime },X_{\gamma }^{*},Z,y∼N\left[\mu_{y}|V_{y}\left\{M^{T}\left(y-Zb\right)/\sigma_{y}^{2}+X_{\gamma }^{*}w_{\gamma }^{*}/\sigma_{\varepsilon }^{2}\right\},V_{y}\right],$$

(15) $$w_{\gamma }^{*}|\theta^{\prime },X_{\gamma }^{*},Z,y∼N\left\{w_{\gamma }^{*}|V_{w_{\gamma }^{*}}\left(X_{\gamma }^{*T}\mu_{y}+\Sigma_{w_{\gamma }^{*}}^{-1}m_{\gamma }\right),\sigma_{\varepsilon }^{2}V_{w_{\gamma }^{*}}\right\},$$

(16) $$b|\theta^{\prime },X_{\gamma }^{*},Z,y∼N\left\{b|\left(1/\sigma_{y}^{2}\right)V_{b}Z^{T}\left(y-M\mu_{y}\right),V_{b}\right\},$$

(17) $$\mu_{w}|\theta^{\prime },X_{\gamma }^{*},Z,y∼N\left\{\mu_{w}|V_{\mu_{w}}\left(1w_{\gamma }/\sigma_{w}^{2}+\mu_{0}/\sigma_{0}^{2}\right),\sigma_{\varepsilon }^{2}V_{\mu_{w}}\right\},$$

(18) $$\sigma_{y}^{2}|\theta^{\prime },X_{\gamma }^{*},Z,y∼IG\{\sigma_{y}^{2}\left|\|y\right\|/2+\alpha_{y},\beta_{y}+\frac{1}{2}{\left(y-M\mu_{y}-Zb\right)}^{T}\left(y-M\mu_{y}-Zb\right)\},$$

(19) $$\sigma_{w}^{2}|\theta^{\prime },X_{\gamma }^{*},Z,y∼IG\{\sigma_{w}^{2}\left|\;\right\|w_{\gamma }\left\|/2+\alpha_{w},\beta_{w}+\left(1/2\sigma_{\varepsilon }^{2}\right){\left(w_{\gamma }-I\mu_{w}\right)}^{T}\left(w_{\gamma }-I\mu_{w}\right)\right\},$$

(20) $$\sigma_{b,\;g}^{2}|\theta^{\prime },X_{\gamma }^{*},Z,y∼IG(\sigma_{b,\;g}^{2}\left|\;\right\|b_{g}\left\|/2+\alpha_{b,\;g},\beta_{b,\;g}+\frac{1}{2}b_{g}^{T}b_{g}\right),$$

(21) $$\sigma_{\varepsilon }^{2}|\theta^{\prime },X_{\gamma }^{*},Z,y∼IG\{\sigma_{\varepsilon }^{2}\left|(\right\|\mu_{y}\left\|+\right\|w_{\gamma }^{*}\|+1)/2+\alpha_{\varepsilon },\beta_{\varepsilon }+\frac{1}{2}R_{\sigma_{\varepsilon }^{2}}\},$$

(22) $$\pi |\theta^{\prime },X_{\gamma }^{*},Z,y∼\beta (\pi |\alpha_{\pi }+\|\gamma \|,\beta_{\pi }+J-\|\gamma |),$$

where we sample $\sigma_{b,\;g}^{2}$ for each *g*. We also define $V_{y}={\left\{\left(1/\sigma_{\varepsilon }^{2}\right)I\;+\;M^{T}M/\sigma_{y}^{2}\right\}}^{-1}$, $V_{w_{\gamma }^{*}}={\left(X_{\gamma }^{*T}X_{\gamma }^{*}+\Sigma_{w_{\gamma }^{*}}^{-1}\right)}^{-1}$, $V_{b}={\left\{\left(1/\sigma_{y}^{2}\right)Z^{T}Z+\Sigma_{b}^{-1}\right\}}^{-1}$, $V_{\mu_{w}}=(1/\sigma_{0}^{2}+\|w_{\gamma }{\left\|/\sigma_{w}^{2}\right)}^{-1}$ and $R_{\sigma_{\varepsilon }^{2}}={\left(\mu_{y}-X_{\gamma }^{*}w_{\gamma }^{*}\right)}^{T}\left(\mu_{y}-X_{\gamma }^{*}w_{\gamma }^{*}\right)+{\left(w_{\gamma }^{*}-m_{\gamma }\right)}^{T}\Sigma_{w_{\gamma }^{*}}^{-1}\left(w_{\gamma }^{*}-m_{\gamma }\right)+{\left(\mu_{w}-\mu_{0}\right)}^{T}\left(\mu_{w}-\mu_{0}\right)/\sigma_{0}^{2}$ for notational simplicity.

Collapsing can lead to improved mixing and convergence, e.g. Andrieu and Doucet (1999). We take advantage of the induced conjugacy to sample the parameters ***γ***, $w_{\gamma }^{*}$, *μ*_*w*_, $\sigma_{\varepsilon }^{2}$ and *π* as a series of collapsed distributions rather than through Gibbs sampling:

(23) $$p\left(\gamma ,w_{\gamma }^{*},\mu_{w},\sigma_{\varepsilon }^{2},\pi \right)=p\left(\gamma \right)p\left(\pi |\gamma \right)p\left(\sigma_{\varepsilon }^{2}|\pi ,\gamma \right)p\left(\mu_{w}|\sigma_{\varepsilon }^{2},\pi ,\gamma \right)p\left(w_{\gamma }^{*}|\mu_{w},\sigma_{\varepsilon }^{2},\pi ,\gamma \right)$$

(24) $$=p\left(\gamma \right)p\left(\pi |\gamma \right)p\left(\sigma_{\varepsilon }^{2}|\gamma \right)p\left(\mu_{w}|\sigma_{\varepsilon }^{2},\gamma \right)p\left(w_{\gamma }^{*}|\mu_{w},\sigma_{\varepsilon }^{2},\gamma \right)$$

where the conditionality on ***θ*** ^′^, **X**,** Z** and **y** has been dropped in the notation and the simplification from equation (23) to equation (24) follows from the conditional independence relationships that are shown in Fig. 2, exploiting the fact that *π* is *d* separated from the remaining parameters in the argument via ***γ***. These distributions can be found by collapsing over parameters as derived in section 1.2 of the on‐line supplementary materials.

### 4.1 Sampling the latent indicators

Sampling ***γ*** is computationally expensive, because it does not naturally taking a distribution of standard form. However, a conditional distribution can still be obtained and Davies *et al*. (2014, 2017) used collapsing methods following Sabatti and James (2005) to achieve faster mixing and convergence as follows:

(25) $$p\left(\gamma |\theta^{\prime },D_{\gamma }^{*},Z,y\right)\propto \int p\left(\gamma ,\pi ,\sigma_{\varepsilon }^{2},w_{\gamma }^{*},\mu_{w}|\theta^{\prime },D_{\gamma }^{*},Z,y\right)d\mu_{w}dw_{\gamma }^{*}d\pi d\sigma_{\varepsilon }^{2}$$

using the likelihood for the conjugate SABRE model given in expression (1) and the same priors that are used for eSABRE. The closed form solution of this integral can be found in the appendix (A.1.5) of Davies (2016).

However, with the likelihood for the conjugate SABRE model given in expression (1) the computational cost of computing expression (25) becomes dependent on inverting a ‖**y**‖×‖**y**‖ matrix. The inversion of this matrix has a computational complexity of *O*(*p* ^2^ *n*) if the Woodbury identity is used, where *p*=‖***γ***‖+1 and *n*=‖**y**‖. This is a result of integrating over $w_{\gamma }^{*}$ to give a multivariate Gaussian distribution of dimension ‖**y**‖. For the size of the data sets that were used in Davies *et al*. (2014, 2017) this is not problematic: ‖**y**‖=246 for example. However, with the H1N1 data set, where ‖**y**‖=15693, calculating any distribution with complexity *O*(*p* ^2^ *n*) becomes less practical.

It is at this point that the structure of the two main probability distributions of eSABRE, expressions (2) and (3), show the huge computational advantage of eSABRE over the conjugate SABRE model that was proposed in Davies *et al*. (2017); see Table 1 in Section 6 for an example of the improved computational efficiency. As in the conjugate SABRE model we use collapsing methods and collapse over $\mu_{w}$, $w_{\gamma }^{*}$, *π* and $\sigma_{\varepsilon }^{2}$. However, whereas the integration over $w_{\gamma }^{*}$ in the conjugate SABRE model gives a multivariate Gaussian distribution of size ‖**y**‖, for eSABRE we instead obtain a multivariate Gaussian distribution of dimension ‖***μ***_**y**_‖ after integrating over $w_{\gamma }^{*}$:

(26) $$p\left(\gamma |\theta^{\prime },X_{\gamma }^{*},\mu_{y}\right)\propto \int p\left(\gamma ,\pi ,\sigma_{\varepsilon }^{2},w_{\gamma }^{*},\mu_{w}|\theta^{\prime },X_{\gamma }^{*},\mu_{y}\right)d\mu_{w}dw_{\gamma }^{*}d\pi d\sigma_{\varepsilon }^{2}$$

where the full distribution can be found in section 1.1 of the on‐line supplementary materials. This dependence on ‖***μ***_**y**_‖ rather than ‖**y**‖ is where the main computational cost reduction occurs, as in the H1N1 data set ‖***μ***_**y**_‖=570 is much smaller than ‖**y**‖=15693. Even with the matrix inversion having a computational complexity of *O*(*p* ^2^ *n*) rather than *O*(*n* ^3^), this still means that the computational complexity of evaluating this density in the SABRE method is 27.5 more than it is in the eSABRE method. It is this reduction in computational costs compared with the SABRE method that makes eSABRE feasible for the H1N1 data set, where the computational cost of the SABRE models is prohibitive.

Multiple methods have been proposed for sampling the latent variables ***γ***. Davies *et al*. (2014) looked at two of these in particular: the componentwise Gibbs sampling approach and a Metropolis–Hastings step (Metropolis *et al*., 1953; Hastings, 1970). In those studies it was found that block Metropolis–Hastings sampling was the method that offered the quickest convergence of the model based on central processor unit (CPU) time and we have therefore used this method here.

Block Metropolis–Hastings sampling improves mixing and convergence through proposing sets *S* of latent indicator variables ***γ***_*S*_ simultaneously, where ***γ***_*S*_ denotes a column vector of all the *γ*_*j*_s where *j* ∈ *S* and ***γ***_−*S*_ its complement. The proposals are then accepted with the following acceptance rate based on the current state c of all the other *γ*s:

(27) $$\alpha \left(\gamma_{S}^{*},\gamma_{S}^{c}|\theta^{\prime },X_{\gamma }^{*},Z,y,\gamma_{-S}^{c}\right):=\min\;\;\left\{\frac{q\left(\gamma_{S}^{c}|\gamma_{S}^{*},\pi \right)p\left(\gamma_{S}=\gamma_{S}^{*},\gamma_{-S}^{c}|\theta^{\prime },X_{\gamma }^{*},Z,y\right)}{q\left(\gamma_{S}^{*}|\gamma_{S}^{c},\pi \right)p\left(\gamma_{S}=\gamma_{S}^{c},\gamma_{-S}^{c}|\theta^{\prime },X_{\gamma }^{*},Z,y\right)},1\right\}$$

where *q*(·) is a proposal density, which we set to be $q\left(\gamma_{S}^{*}|\gamma_{S}^{c},\pi \right)=\Pi_{j\in S}\text{Bern}\left(\gamma_{j}^{*}|\pi \right)$. For SABRE expression (25) is used for computing *p*(·) in expression (27), whereas expression (26) is used for eSABRE. Proposed moves for independent sets of randomly ordered inclusion parameters $\gamma_{S}^{*}$ are then accepted if $\alpha (\gamma_{S}^{*},\gamma_{S}^{c}|\theta^{\prime },X_{\gamma }^{*},Z,y,\gamma_{-S}^{c})$ is greater than a random variable u∼U[0,1], until updates have been proposed for all the latent indicator variables. The size of the proposal sets, *S*, was investigated in detail in Davies *et al*. (2014, 2017) and we have followed those guidelines here when choosing the size of *S*.

## 5 Model selection for choosing the random‐effect components

There are various methods that can be used to select the random effects that should be used within a model. Previously Davies *et al*. (2016) compared tenfold Bayesian CV and WAIC (Watanabe, 2010), and found that in terms of model selection WAIC achieved a similar performance at a lower computational cost to tenfold Bayesian CV. Here we look at Bayesian integrated CV (ICV), e.g. Vehtari and Ojanen (2012), and several variations of WAIC that can be applied to latent variable models.

An alternative approach to those suggested above would be to use spike‐and‐slab priors to select the random effects. Although this would require only one model to be fitted, doing so will come at a large computational cost. This is a result of poor mixing that is associated with proposing MCMC moves which change entire groups of random‐effect coefficients simultaneously. Using intramodel approaches for a small number of models in parallel is far more computationally viable and has therefore been used here. We have further discussed the decision to choose WAIC‐based methods over spike‐and‐slab prior based methods for the selection of random‐effect components in section 3 of the on‐line supplementary materials.

### 5.1 Integrated cross‐validation

Bayesian CV methods are reliable, if computationally expensive, techniques for measuring the out‐of‐sample performance of different models. Bayesian ICV, e.g. Vehtari and Ojanen (2012), is a special version of CV which works well in latent variable models. Bayesian ICV integrates over the latent variables, in this case ***μ***_**y**_, to give the following utility function for *k*‐fold Bayesian ICV:

(28) $$p_{\mathrm{ICV}}=\frac{1}{K}\sum_{k=1}^{K}\log\;\;\left(\frac{1}{I}\right)\sum_{\iota =1}^{I}N\left(y_{k}|M_{k}X_{\gamma ,\;k}^{*}w_{\gamma }^{*,\;\iota }+Z_{k}b^{\iota },\sigma_{y}^{\iota ,\;2}I_{k}+\sigma_{\varepsilon }^{\iota ,\;2}M_{k}M_{k}^{T}\right)$$

where the **y**_*k*_, $X_{\gamma ,\;k}^{*}$ and **Z**_*k*_ are the held‐out data for validation and there are *I* iterations of the MCMC scheme. The distribution comes from integrating over ***μ***_**y**_ in the distribution given by the product of distributions (2) and (3). The parameter samples, $w_{\gamma }^{*,\;\iota }$, $b^{\iota }$, $\sigma_{y}^{\iota ,\;2}$ and $\sigma_{\varepsilon }^{\iota ,\;2}$ for *ι* ∈ {1,…,*I*}, are taken by using the MCMC algorithm to sample from the posterior of eSABRE applied to **y**_−*k*_, **X**_−*k*_, **Z**_−*k*_ and **M**_−*k*_.

### 5.2 Block integrated WAIC

WAIC, as proposed in Watanabe (2010), is natural for selecting the correct model when the underlying model is singular, i.e. models with a non‐identifiable parameterization, such as SABRE and eSABRE. WAIC has been proven to be asymptotically equivalent to Bayesian leave‐one‐out CV (LOOCV) in Watanabe (2010) and is computed as follows from posterior samples of the model parameters $\theta^{\iota }$ for *ι* ∈ {1,…,*I*}:

(29) $$p_{\mathrm{WAIC}}=-2\sum_{i=1}^{N}\left(\;\log\;\;\left\{\frac{1}{I}\sum_{\iota =1}^{I}p\left(y_{i}|\theta^{\iota },X_{\gamma ,\;i},Z_{i}\right)\right\}-\text{var}\left[\log\left\{p\left(y_{i}|\theta^{\iota },X_{\gamma ,\;i},Z_{i}\right)\right\}\right]\right),$$

where var is the sample variance with respect to $\theta^{\iota }$, and **X**_*i*_ and **Z**_*i*_ are the *i*th row of the fixed and random‐effects design matrices respectively. WAIC can be used for a wide variety of problems; however, it is only justifiable for problems where the observed data are independently distributed with a population distribution, e.g. SABRE where the joint likelihood is given by expression (1). The inclusion of latent variables in eSABRE means that the observed data are not modelled with independent distributions and it is therefore inaccurate to use WAIC with eSABRE.

To make WAIC more applicable to latent variable models such as eSABRE, Li *et al*. (2016) introduced two alternative versions of WAIC: non‐integrated WAIC, nWAIC, and integrated WAIC, iWAIC. nWAIC applies WAIC to the predictive density of the observed variables **y**=(*y*_1_,…,*y*_*N*_), conditionally on the model parameters ***θ*** and the potentially correlated latent variables ***ψ***=(*ψ*_1_,…,*ψ*_*N*_):

(30) $$p_{\mathrm{nWAIC}}=-2\sum_{i=1}^{N}\left(\;\log\;\;\left\{\frac{1}{I}\sum_{\iota =1}^{I}p\left(y_{i}|\theta^{\iota },\psi_{i}^{\iota },Z_{i}\right)\right\}-\text{var}\left[\log\left\{p\left(y_{i}|\theta^{\iota },\psi_{i}^{\iota },Z_{i}\right)\right\}\right]\right)$$

where $\theta^{\iota }$ and $\psi_{i}^{\iota }$ are sampled from the posterior distribution via MCMC sampling and var is the sample variance. In the proposed eSABRE, taking just the likelihood for *y*_*i*_ from distribution (2) would be the distribution corresponding to $p(y_{i}|\theta^{\iota },\psi_{i}^{\iota },Z_{i})$ and would not satisfy the independence assumptions of WAIC‐based methods as each *y*_*i*_ is dependent on a latent variable *ψ*_*i*_ which is shared by other observations.

Only using the likelihood of the model, e.g. distribution (2), in equation (30) also means that nWAIC does not account for the mismatch in the model fit of the latent variables as they are described in distribution (3). This means that nWAIC does not take into account how well the latent variables are predicted by the explanatory variables. Li *et al*. (2016) therefore proposed iWAIC:

(31) $$p_{\mathrm{iWAIC}}=-2\sum_{i=1}^{N}\left(\;\log\;\left\{\frac{1}{I}\sum_{\iota =1}^{I}p\left(y_{i}|\theta^{\iota },X_{\gamma ,\;i},Z_{i},\psi_{-i}^{\iota }\right)\right\}-\text{var}\left[\log\left\{p\left(y_{i}|\theta^{\iota },X_{\gamma ,\;i},Z_{i},\psi_{-i}^{\iota }\right)\right\}\right]\right)$$

where var is the sample variance and the distribution that is used is given by $p(y_{i}|\theta^{\iota },X_{\gamma ,\;i},Z,\psi_{-i}^{\iota })$ $=\int p\left(y_{i}|\theta^{\iota },\psi_{-i}^{\iota },\psi_{i},Z\right)p\left(\psi_{i}|\theta^{\iota },X_{\gamma }\right)d\psi_{i}$, the analytical integration of the latent variables from the product of the likelihood and the distribution of the latent variables.

The proposed version of iWAIC does not, however, work with eSABRE. This is because each observation *y*_*i*_ does not have its own corresponding latent variable *ψ*_*i*_. Instead any two observations *y*_1_ and *y*_2_ from the same pair of reference and test viruses, *p*, will have the same latent variable, i.e. *ψ*_1_=*ψ*_2_=*μ*_**y**,*p*_. Under this model, i.e. where *ρ*(*ψ*_1_,*ψ*_2_)=1, it is mathematically intractable to integrate over *ψ*_1_=*μ*_**y**,*p*_ without integrating over *ψ*_2_=*μ*_**y**,*p*_, something which is required to calculate $p(y_{i}|\theta^{\iota },X_{\gamma ,\;i},Z_{i},\psi_{-i})$ as needed for equation (31). We must therefore either use nWAIC given by equation (30) or find an alternative.

In this current work we propose biWAIC, which is a new modification of WAIC for latent variable models with latent variables that are either completely correlated or have no correlation. In the eSABRE method we use the latent variables ***μ***_**y**_, where it is possible for two observations that have the same latent variables, e.g. *ψ*_1_=*ψ*_2_=*μ*_**y**,*p*_, in replacement of ***ψ***. Unlike WAIC, nWAIC and iWAIC, which rely on using independent distributions for each *y*_*i*_, biWAIC instead uses a distribution for independent groups of observations **y**_*p*_ with the same associated latent variable. In this way, **y**_*p*_ is the group containing all *y*_*i*_ whose virus pair *p*_*i*_ is the same as the group's virus pair *p*. Given this specification of groups, it is then possible to integrate analytically over the corresponding latent variable *μ*_*y*,*p*_ of the product of the likelihood and the distribution of the latent variables taken from distributions (2) and (3): $p\left(y_{p}|\theta^{\iota },X_{\gamma ,\;p},Z\right)=\int p\left(y_{p}|\theta^{\iota },\mu_{y,\;p},Z\right)$ $p\left(\mu_{y,\;p}|\theta^{\iota },X_{\gamma ,\;p}\right)d\mu_{y,\;p}$. biWAIC can then be written as

(32) $$p_{\mathrm{biWAIC}}=-2\sum_{p=1}^{P}\left(\;\log\;\left\{\frac{1}{I}\sum_{\iota =1}^{I}p\left(y_{p}|\theta^{\iota },X_{\gamma ,\;p},Z_{p}\right)\right\}-\text{var}\left[\log\left\{p\left(y_{p}|\theta^{\iota },X_{\gamma ,\;p},Z_{p}\right)\right\}\right]\right)$$

where var is the sample variance.

As well as being applicable to eSABRE and particular specifications of latent variable models, biWAIC can also be shown to have some useful asymptotic properties. Previously Watanabe (2010) has shown that WAIC is asymptotically equivalent to Bayesian LOOCV, based on the fact that Bayesian LOOCV loss is asymptotically equivalent to WAIC as a random variable. Although biWAIC is not asymptotically equivalent to Bayesian LOOCV, on the basis of the same proof of Watanabe (2010) we can determine that it is asymptotically equivalent to a different form of Bayesian CV. From looking at equations (28) and (32), along with the two distributions from which those equations are derived (distributions (2) and (3)), we can see that, if ICV is evaluated on the same groups as biWAIC, then it is asymptotically equivalent to biWAIC as a random variable. biWAIC is therefore asymptotically equivalent to Bayesian leave one group out CV, where observations are divided into *P* independent groups based on the number of different virus pairs (groups), as opposed to *n* groups of single observations for Bayesian LOOCV.

### 5.3 Summarizing remarks

In summary, we have discussed ICV, e.g. Vehtari and Ojanen (2012), which is a version of CV that is designed for latent variable methods but is computationally expensive. A computationally cheaper alternative is WAIC and this has been shown to give similar performance in some cases (Davies *et al*., 2016). However, standard WAIC is not appropriate for latent variable models such as the eSABRE method as it assumes independence between observations. nWAIC, proposed in Li *et al*. (2016), naively applies WAIC to the likelihood of the model, distribution (2), but does not take into account the fit of the latent variables, distribution (3). As an improvement, Li *et al*. (2016) also proposed iWAIC for latent variable models, but this is not suitable for the eSABRE method as each observation *y*_*i*_ does not have an individual latent variable *ψ*_*i*_. Instead we propose biWAIC which allows for the latent variable structure of the eSABRE method and takes into account both distribution (2) and distribution (3) by effectively applying iWAIC at a group level rather than an individual level.

## 6 Simulation studies

### 6.1 Simulated data sets

In this section we describe the simulated data sets that are used to test the effectiveness of eSABRE proposed here and compare it with the conjugate SABRE model that was described in Davies *et al*. (2016).

#### 6.1.1 Influenza‐inspired simulated data

To test initially eSABRE and the conjugate SABRE model we generated three data sets with a reasonably small number of variables. These three data sets are based on the same structure as the influenza data sets with a varied number of random‐effect factors. In each of the data sets 2000 observations were simulated from 55 pairs of viruses. The 55 pairs of viruses come from having 10 viruses tested against each other ($(\begin{array}{c} 10\\ 2 \end{array})=45$) plus the viruses tested against themselves (expression (10)), with each of these pairs then given 50 possible fixed effects and four possible random‐effect components (including the reference and test viruses). The random‐effects groups were included with probability 0.5 and given zero coefficients otherwise, whereas the relevant coefficients were generated from a zero‐mean Gaussian distribution with each component having a fixed variance drawn from *U*(0.2,0.5). Fixed effects *w*_*j*_ were given non‐zero values generated from a uniform distribution, *U*(−0.4,−0.2), with inclusion probability *π*∼*U*(0.2,0.4). $\sigma_{y}^{2}$ and $\sigma_{\varepsilon }^{2}$ were both set to be 0.033, 0.1 and 0.3 for the three simulated data sets.

#### 6.1.2 Foot‐and‐mouth disease virus simulated data

To make the simulation studies more realistic we wanted to make simulated data sets based on the influenza A(H1N1) data set that was described in Section 2.1. However, although this does not cause any problems for the proposed eSABRE model, using the conjugate SABRE model to analyse data sets of this size is computationally prohibitive. Therefore instead we have created 20 simulated data sets based on the extended South African Territories type 1 FMDV data set that was used in Reeve *et al*. (2016) and Davies *et al*. (2017). These data sets were created to be the same size as the FMDV data sets by using the maximum *a posteriori* parameter estimates of the eSABRE method applied to the South African Territories type 1 FMDV data set. However, to highlight the differences in performance of the two models under different circumstances, we varied the error of the underlying model, $\sigma_{\varepsilon }^{2}\in \left\{0.02,0.2,0.5\right\}$, and changed the mean of the regression parameters, $\mu_{w}\in \left\{-0.1,-0.3,-0.5\right\}$. Following Reeve *et al*. (2016) we used three random‐effect components: the test virus, the date of the experiment and the antiserum (reference virus).

#### 6.1.3 Simulated data for model selection

Finally, to compare nWAIC, biWAIC and tenfold Bayesian ICV, we have generated nine sets of 20 data sets with up to four random effects: the test virus, the reference virus and two generic random‐effect factors. The data sets were generated with 50 possible fixed effects and up to four random‐effect factors included with probability 0.5. Of the nine sets of data sets, three contain 10 virus strains, where each virus strain has been used as both a reference and a test virus, meaning that there are 55 pairs of reference and test viruses; see Section 6.1.1. Following the same set‐up, three of the sets of data sets include 30 virus strains (465 pairs) and the other three have 45 virus strains (1035 pairs). Within each of these sets of three data sets, the model error $\sigma_{\varepsilon }^{2}$ was varied to be either 0.1, 0.3 or 0.5.

### 6.2 Computational inference

For the simulated data we generated 10000 parameter samples from the model, removing 2000 for burn‐in. We have previously explored speed of parameter convergence extensively in Davies *et al*. (2014, 2017). In that work we established the amount of samples required to achieve convergence based on using four chains and potential scale reduction factors PSRF (Gelman and Rubin, 1992). For this we took the threshold of convergence to be PSRF⩽1.1 and terminated the burn‐in phase when this was satisfied for 95% of the variables. Given that the trace plots and other diagnostic plots indicate that the model parameter profiles give similar parameter sample trajectories characteristics to those tested in Davies *et al*. (2014, 2017), we have used the MCMC specification as established in Davies *et al*. (2014, 2017) to reduce the computational requirements of the multiple simulation studies that we have implemented. An example of the computational requirements to do this is provided in Table 1, part (b).

For the H1N1 data set we generated four chains of parameter samples and computed the PSRFs. We took the threshold of convergence to be PSRF⩽1.1 and terminated the burn‐in phase when this was satisfied for 95% of the variables.

The fixed hyperparameters, which are shown as grey nodes in Fig. 2, were set the same for both the eSABRE and conjugate SABRE methods such that ***α***_**b**_=***β***_**b**_=(0.001,…,0.001), *α*_*w*_=*β*_*w*_=*α*_*y*_=*β*_*y*_=*α*_*ɛ*_=*β*_*ɛ*_=0.001, *μ*_0_=0, $\sigma_{0}^{2}=100$, $w_{0}=\max\left(y\right)$, *α*_*π*_=1 and *β*_*π*_=4 following Davies *et al*. (2017).

### 6.3 Results for the simulation studies

Table 1, part (a), gives the area under the receiver operating characteristic curve values AUC for eSABRE proposed here and the conjugate SABRE model that was described in Davies *et al*. (2017) applied to the influenza‐inspired simulated data sets from Section 6.1.1. The AUC‐values are calculated on the basis of the correct selection or exclusion of the various fixed effects in the model, where variables are ranked on the basis of the proportion of times that they are selected in the model. For each combination of data set and number of observations, eSABRE offers a clear improvement in terms of global variable‐selection performance over SABRE. This improvement is a result of the latent variable structure of eSABRE which better reflects the data generation process, where the difference in the models can be seen by comparing the probabilistic graphical models in Fig. 2 here and Fig. 1 in Davies *et al*. (2017). Table 1, part (a), shows how this improvement is more significant as the effect of the latent variable structure is increased, i.e. as $\sigma_{\varepsilon }^{2}$ is increased. As the error variances grow larger, e.g. 0.1 and 0.3, eSABRE offers a much clearer improvement over the conjugate SABRE model than it did it in the data set where the error variances are smaller: 0.033. This is because the conjugate SABRE model and eSABRE become more similar as $\sigma_{\varepsilon }^{2}\to 0$. Given the large variance in HI titre measurements (0.23 (to two decimal places) for the H1N1 data), for any given pair of reference and test viruses in the H1N1 data set, this improvement is vital. Additionally we have shown that the eSABRE method outperforms the conjugate SABRE method in terms of out‐of‐sample prediction: see Table 1 in the on‐line supplementary materials.

Another notable result from Table 1, part (a), is the reduction in performance in terms of AUC‐values of the conjugate SABRE model as the number of observations increases. This is an unexpected result as we would expect more data to provide more information to the model, resulting in a better selection of variables in the models and higher AUC‐values. The reason for this unexpected result is a consequence of the mismatch between the data generation process where variance in the observations comes in two forms, $\sigma_{\varepsilon }^{2}$ and $\sigma_{y}^{2}$, and the model which only directly accounts for the variance in **y** given by $\sigma_{y}^{2}$.

To demonstrate that the unexpected reduction in performance of the conjugate SABRE model is a result of the mismatch between the data and the model we completed a small simulation study with linear models. We generated groups of data sets with 500, 1000 and 2000 observations generated from a linear model with each group containing 2000 data sets. For each of these groups, half the data sets have observations generated with independent and identically distributed noise, e.g. $y∼N(y|Xw,\sigma_{y}^{2}I)$. The other half of the data sets were given correlated errors based on integrating over a set of random effects, e.g. $y∼N(y|Xw,\sigma_{y}^{2}I+\sigma_{\varepsilon }^{2}MM^{T})$. This is equivalent to integrating over the latent variables but allows us to use the same **X** and **w** for a fair comparison. Additionally each of the data sets contains two variables, one relevant, **x**_r_, and one irrelevant, **x**_ir_. We then calculated the marginal likelihood for each of the four possible models

- no variables included, *p*(**y**|·),
- irrelevant variable included only, *p*(**y**|**x**_ir_),
- relevant variable included only, *p*(**y**|**x**_r_), and
- both variables included, *p*(**y**|**x**_ir_,**x**_r_),

under the assumption of IID noise, as the conjugate SABRE model assumes (incorrectly) with the H1N1 data, where we have fixed $\sigma_{w}^{2}$ and marginalized out $\sigma_{y}^{2}$ and **w**. We can then use these marginal likelihoods to calculate the probability that the irrelevant variable is included in the final model M via Bayes theorem as follows:

(33) $$P\left(x_{\mathrm{ir}}\in M\right)=\frac{p\left(y|x_{\mathrm{ir}}\right)+p\left(y|x_{\mathrm{ir}},x_{r}\right)}{p\left(y|\cdot \right)+p\left(y|x_{\mathrm{ir}}\right)+p\left(y|x_{r}\right)+p\left(y|x_{\mathrm{ir}},x_{r}\right)}.$$

**Figure 3:**
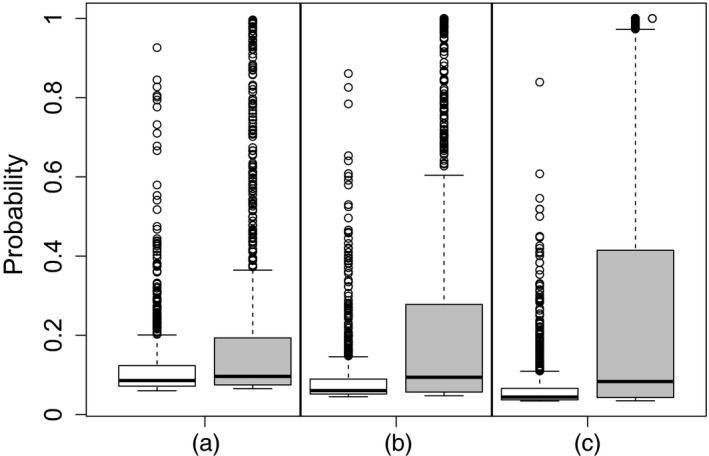
Boxplots showing the effect of non‐IID Gaussian noise on a model assuming IID Gaussian noise (the boxplots show the probability that an irrelevant variable is included in a model for data with IID Gaussian noise (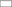) against the probabilities for a model with a noise structure based on the H1N1 data set (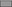); the results show the probability that the irrelevant variable is included in the model decreases as the number of observations increases for the data with IID Gaussian noise; conversely it shows an increase in the probability of its inclusion as the number of observations increases when there is a noise structure based on the H1N1 data set): (a) 500 observations; (b) 1000 observations; (c) 2000 observations

Fig. 3 gives boxplots of the probability that the irrelevant variable **x**_ir_ is included in the final model for each of the data sets from our small simulation study. The boxplots show the effect on the probabilities caused by the different types of noise and varied amounts of observations. Fig. 3 shows that, as the number of observations increases, the chance that the irrelevant variable will be included decreases for the IID noise, as would be expected. However, for the non‐IID noise based on the FMDV and influenza data sets, the results show an increase in the probability that the irrelevant variable will be included as the number of observations increases, indicating that the model mismatch that is inherent in SABRE is what causes the unexpected results in Table 1, part (a).

Table 1, part (b), shows the improvement that eSABRE offers over the conjugate SABRE model in terms of computational efficiency. Table 1, part (b), shows how the conjugate SABRE model becomes far more computationally expensive as the number of observations increases, whereas the required CPU time hardly changes for eSABRE if the number of pairs of reference and test viruses remains the same. This improvement in terms of computational efficiency explains why it is viable to use eSABRE on the H1N1 data set for example, where ‖*y*‖=15693 and *P*=570, but not the conjugate SABRE model that was described in Davies *et al*. (2017).

Table 2 shows the effectiveness of eSABRE on larger more realistic data sets (Section 6.1.2) based on the real life FMDV data from Reeve *et al*. (2016). Like Table 1 the results of Table 2 again show that eSABRE clearly outperforms the conjugate SABRE model in all scenarios from Section 6.1.2, except for the data set where $\mu_{w}=-0.1$ and $\sigma_{\varepsilon }^{2}$. The results show that, as the model error in the simulated data increases, the conjugate SABRE model seriously drops off in performance whereas eSABRE remains reasonably consistent. Like with the results of Table 1, the difference in performance is again caused by the mismatch between the conjugate SABRE model and the underlying data generation process. As $\sigma_{\varepsilon }^{2}$ increases, the SABRE method matches the data generation process less, whereas eSABRE can model this change in value.

**Table 2:** Table of AUC‐values for eSABRE and the conjugate SABRE model when applied to the FMDV‐based simulated data sets†

$\mu_{w}$	*Results for eSABRE*			*Results for conjugate SABRE*		
	$\sigma_{\varepsilon }^{2}=0.02$	$\sigma_{\varepsilon }^{2}=0.2$	$\sigma_{\varepsilon }^{2}=0.5$	$\sigma_{\varepsilon }^{2}=0.02$	$\sigma_{\varepsilon }^{2}=0.2$	$\sigma_{\varepsilon }^{2}=0.5$
−0.1	0.67	0.67	0.63	0.69	0.60	0.57
−0.3	0.72	0.70	0.67	0.71	0.61	0.58
−0.5	0.75	0.74	0.73	0.72	0.64	0.57

† The table gives AUC‐values for eSABRE and the conjugate SABRE model, when applied to the FMDV‐based simulated data sets described in Section 6.1.2.

**Figure 4:**
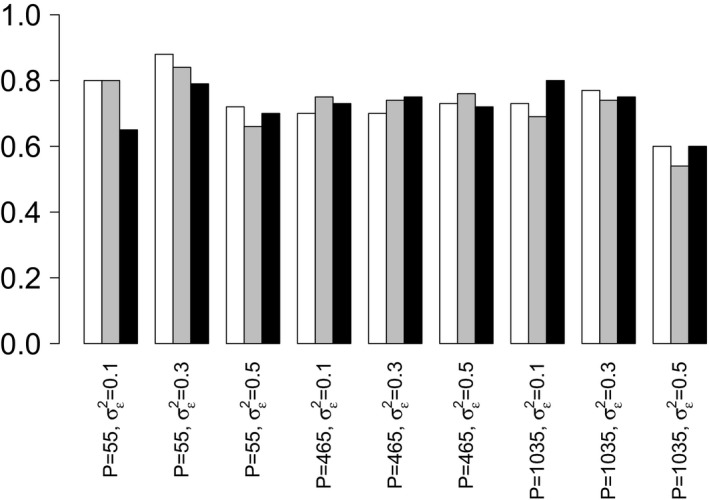
Bar plot of F1‐scores given in Table 3: the bar plot compares the F1‐scores for nWAIC (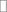), biWAIC (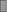) and Bayesian tenfold ICV (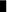) in terms of correctly selecting random‐effect components for the data set described in Section 6.1.3; the figure takes the results from Table 3

**Figure 5:**
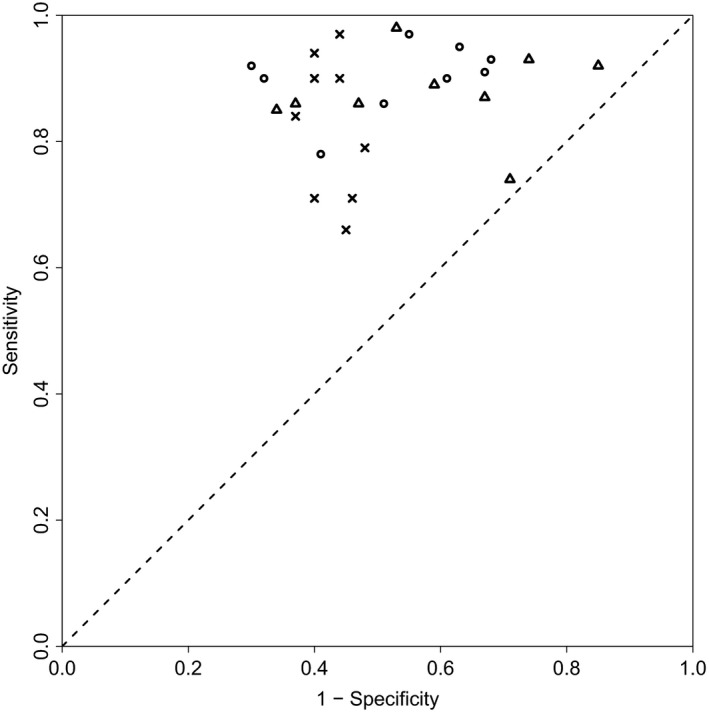
Plot of sensitivities and 1 minus specificities for the results given in Table 3: the plot compares nWAIC (∘), biWAIC (×) and Bayesian tenfold iCV (▵) in terms of correctly selecting random‐effect components for the data set described in Section 6.1.3; the figure takes the results from Table 3 and plots the sensitivities against the complementary specificities (i.e. 1 minus specificities), i.e. as single points from a receiver operating characteristic curve

To compare the methods that were described in Section 5, nWAIC, biWAIC and Bayesian tenfold ICV, we have looked at their performance in terms of correctly selecting random‐effect factors on the data sets from Section 6.1.3. The results are given in Table 3 and are displayed visually in Figs 4 and 5.

**Table 3:** Table of results looking at the random‐effects factor selection performance of the methods described in Section 5†

	*P*	$\sigma_{\varepsilon }^{2}$	*nWAIC*	*biWAIC*	*Bayesian*
					*tenfold ICV*
*Sensitivity*	55	0.1	0.90	0.97	0.92
	55	0.3	0.92	0.90	0.89
	55	0.5	0.78	0.71	0.93
	465	0.1	0.97	0.94	0.85
	465	0.3	0.86	0.84	0.86
	465	0.5	0.95	0.90	0.86
	1035	0.1	0.93	0.71	0.98
	1035	0.3	0.91	0.79	0.87
	1035	0.5	0.90	0.66	0.74
*Specificity*	55	0.1	0.68	0.56	0.15
	55	0.3	0.70	0.60	0.41
	55	0.5	0.59	0.54	0.26
	465	0.1	0.45	0.60	0.66
	465	0.3	0.49	0.63	0.63
	465	0.5	0.37	0.56	0.53
	1035	0.1	0.32	0.60	0.47
	1035	0.3	0.33	0.52	0.33
	1035	0.5	0.39	0.55	0.29
*F1‐score*	55	0.1	0.80	0.80	0.65
	55	0.3	0.88	0.84	0.79
	55	0.5	0.72	0.66	0.70
	465	0.1	0.70	0.75	0.73
	465	0.3	0.70	0.74	0.75
	465	0.5	0.73	0.76	0.72
	1035	0.1	0.73	0.69	0.80
	1035	0.3	0.77	0.74	0.75
	1035	0.5	0.60	0.54	0.60

† The table gives results in terms of the successful selection or exclusion of random‐effects factors when using the methods described in Section 5, nWAIC, biWAIC and Bayesian tenfold ICV, on parameter samples from the posterior distribution of eSABRE applied to the simulated data from Section 6.1.3, where *P* is the number of pairs of reference and test strains. The results are given as sensitivities, specificities and F1‐scores, which are calculated based on the correct inclusion or exclusion of random‐effects factors. The results are displayed in an alternative manner in Figs 4 and 5. F1‐scores, sensitivity and specificity are defined in the text.

The results in Table 3 show that all of the methods, nWAIC, biWAIC and Bayesian tenfold ICV, perform similarly in terms of their overall accuracy in correctly including or excluding random‐effects factors. The similarity is best demonstrated by looking at the F1‐scores, which consider both precision and recall, offering a more general assessment of performance than looking at them separately. (F1‐score=2(precision×recall)/(precision+recall),sensitivity=recall=TP/(TP+FN),specificity=TN/(TN + FP) and precision=TP/(TP + FP), where we define the following parameters: true positive TP, false positive FP, true negative TN and false negative FN.) The F1‐scores from Table 3 can also be seen in Fig. 4 where the results are shown as bar plots. With the results from Table 3 and Fig. 4 suggesting that the information criteria nWAIC and biWAIC give similar selection performances to Bayesian tenfold ICV, it is reasonable to use one of the former criteria on the influenza data set in Section 7, where Bayesian tenfold ICV will be computationally onerous. Additionally we find that there is no difference in terms of out‐of‐sample performance between the models that are selected by nWAIC and biWAIC: see Table 2a in the on‐line supplementary materials.

Although suggesting that the methods perform similarly overall in terms of F1‐scores, Table 3 also indicates that the methods operate with different sensitivity *versus* specificity trade‐offs, meaning that on average some methods include more random‐effect factors than others. This can be seen by looking at the sensitivities and specificities of nWAIC, biWAIC and Bayesian tenfold ICV in Table 3 or alternatively by looking at Fig. 5. Fig. 5 plots the sensitivities that are achieved by the various methods on each set of data sets against the complementary specificity (i.e. 1 minus specificity) and shows that the biWAIC‐method operates at a higher threshold for inclusion, meaning that it selects less random‐effect factors in the model on average. This can be seen by noting the lower sensitivities and higher specificities in Fig. 5 or Table 3.

The reason for the difference between nWAIC and biWAIC in terms of the average number of random‐effect factors that are included is a result of the distribution from which they measure the sample means and variances that are needed to calculate the criterion. nWAIC, equation (30), takes its sample means and variances on the basis of only the distribution of **y**, distribution (2), the distribution which contains the random‐effects specification. biWAIC, expression (32), however, takes its sample means and variances from the marginalized distribution of **y** where ***μ***_**y**_ has been integrated out as detailed in Section 5.2. As a result, like Bayesian tenfold ICV, biWAIC takes into account the model fit of both **y** and ***μ***_**y**_. It is interesting, however, that this does not appear to affect the number of fixed effects that are included in the model: see Table 2b in the on‐line supplementary materials.

Taking into account both of the probability distributions that are associated with the latent variables (distributions (2) and (3)) better assesses the fit of the model and prevents the overfitting of the first distribution (distribution (2)), keeping the number of included random‐effect groups at a realistic level. nWAIC does not take into account how well the fixed effects **w**_***γ***_ can predict the unknown true HI titres of given pairs of reference and test strains, ***μ***_**y**_. nWAIC therefore picks the model which maximizes the fit of distribution (2) regardless of the fit of distribution (3), leading to the overfitting of distribution (2) and a potentially unrealistically high number of random‐effect groups. It is interesting, however, that we do not see a similar threshold with Bayesian tenfold ICV, which also takes into account both parts of the latent variable likelihood. This is a consequence of the different sensitivity *versus* specificity trade‐offs that are given by criteria based on WAIC and those based on CV.

## 7 Results for the A(H1N1) data set

We applied eSABRE to the influenza A(H1N1) data set that was described in Section 2.1, using the eight possible combinations of random‐effect components. (Each application of the model took around 2 days on a standard desktop computer.) The biWAIC‐score, Section 5.2, was then calculated for each of the models, with the model with the best biWAIC‐score including all random‐effect components. biWAIC was chosen to select the best model on the basis of being more computationally feasible than tenfold ICV and the results of Table 3 and Figs 4 and 5.

Having selected the model with the best selection of random‐effect components according to biWAIC, we then compared the results in terms of variable selection with those achieved by Harvey *et al*. (2016). (Additional plots looking at the goodness of fit of the model selected can be found in Figs 1 and 2 of the on‐line supplementary materials.) Using eSABRE as an exploratory tool, where we chose a relatively low threshold by taking the top $\hat{\pi }J$ and variables with the highest marginal inclusion probabilities, we selected 11 proven, three plausible and three implausible residues based on the classifications that were discussed in Section 2.1. ($\hat{\pi }$ is the maximum *a posteriori* estimate of the model parameter *π*. *J* is the number of variables. We estimate $\hat{\pi }\approx 0.18$ and have *J*=275. We therefore select 48 variables, 17 of which are residue variables and 31 are branch variables.) However, for a fair comparison with the results of Harvey *et al*. (2016), we also used a more conservative cut‐off, selecting only variables with a marginal inclusion probability of greater than 0.5, which gives the same number of implausible residues selected. Here we have selected six proven, two plausible and one implausible, compared with four proven, no plausible and one implausible selected in Harvey *et al*. (2016). These results, which are summarized in Table 4, show a clear improvement for eSABRE over the models that were used in Harvey *et al*. (2016).

**Table 4:** Summary of H1N1 results†

*Method*	*Residue classification*		
	*Proven*	*Plausible*	*Implausible*
Harvey *et al*. (2016)	4	0	1
eSABRE (top $\hat{\pi }J$ variables)	11	3	3
eSABRE (inclusion probability >0.5)	6	2	1

† The table shows the number of proven, plausible and implausible residues selected by Harvey *et al*. (2016), the eSABRE method selecting the top $\hat{\pi }J$ variables and the eSABRE method selecting any variable with a marginal inclusion probability of greater than 0.5.

**Figure 6:**
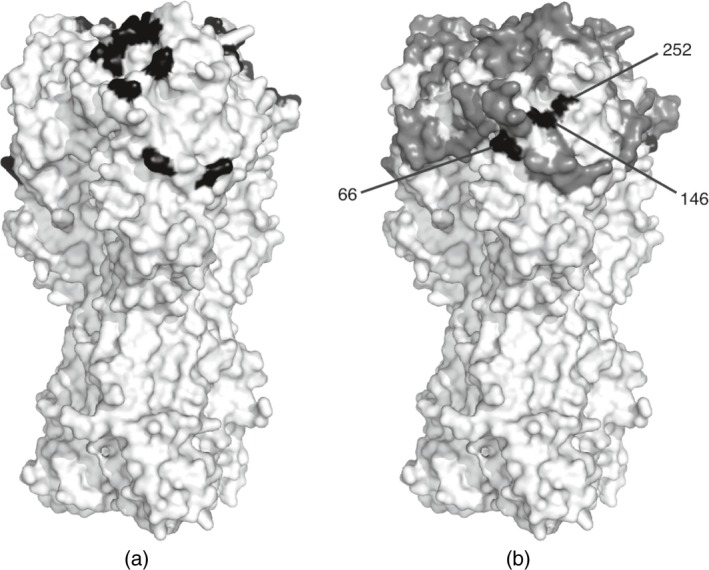
Three‐dimensional structure of the influenza A(H1N1) haemagglutinin protein showing the positions of proven and plausible antigenic residues identified by using eSABRE: (a) proven residues (black) selected by eSABRE; (b) labelled plausible residues (black) where the biologically proven sites from Fig. 1 are shown in dark grey; the representation of the surface of haemagglutinin is based on the resolved structure of influenza A(H1N1) strain A/Puerto Rico/8/34 (Gamblin *et al*., 2004)

Of the 11 proven residues selected and shown in Fig. 6(a), eSABRE, taking the top $\hat{\pi }J$ variables with the highest marginal inclusion probabilities, has identified one residue of the receptor binding site: residue 187. Residue 187 is also part of the Sb antigenic site and we also identified several other nearby residues (184, 189, 190 and 193) belonging to the same antigenic site. We also identified proven residues from each of the other known H1 antigenic sites; Ca (141 and 142), Cb (69 and 74) and Sa (153). Finally we identified the residue at position 130 to be important, which is a proven antigenic residue outside these antigenic sites. An amino acid deletion at this site has been determined to have altered the structure of protein, causing a major change in the antigenic properties of the virus which required an update to the vaccine in the 1990s (McDonald *et al*., 2007).

The three plausible residues that were identified (66, 146 and 252) can be found between the Ca and Cb antigenic sites as can be seen in Fig. 6(b). Fig. 6(b) shows the proven antigenic sites of the A(H1N1) virus (dark grey) and the locations of the plausible residues (black) between them. Although all the plausible residues could be plausibly antigenic, the most likely to be significant are 66 and 146, which both contact known antigenic sites on the surface of the protein. Residue 66 occurs directly between and immediately neighbouring two sections of the Ca and Cb antigenic sites. Both 146 and 252 also occur between sections of the Ca and Cb antigenic regions; however, residue 146 is in a small indent on the surface and so, although remaining very plausible, is perhaps less likely than 66. Residue 252 is further away from the antigenic sites while still remaining in the head region of the virus close to known antigenic sites and was also picked up by Harvey *et al*. (2016).

Of the three implausible residues that were identified (43, 310 and 313), residue 43 has a reasonable explanation why it was selected. Substitutions to the residue at position 43 are known to have occurred at the same branch of the phylogenetic tree as the important deletion that was referred to above at position 130, the proven antigenic residue selected by eSABRE described by McDonald *et al*. (2007), and in a second branch of the tree associated with a vaccine update. The by‐chance co‐occurrence of substitutions at residue 43 and genuine antigenic changes at multiple instances in the evolution of the virus provide an explanation why 43 was identified both here and by using other methods (Harvey *et al*., 2016). The other two implausible residues that were identified, 310 and 313, are part of the stalk domain of the H1N1 virus and are unlikely to have a significant antigenic effect. It is, however, noteworthy that residue 313 was identified at a later, non‐comparable stage of the analysis of Harvey *et al*. (2016) which involved the identification of specific amino acid substitutions that correlated with points in the evolution of the virus where the antigenicity changed.

## 8 Conclusions and future work

We have developed a novel hierarchical Bayesian model, called eSABRE, for detecting antigenic sites in virus evolution, with particular focus on the influenza virus. Our model is based on a predecessor, called SABRE, that was developed in the context of studying antigenicity in the FMDV. However, SABRE turned out to be computationally too inefficient for larger data sets, as are typically available for the influenza virus. We have demonstrated that, by building a new structure of the hierarchical model, we can not only improve the computational efficiency by several orders of magnitude, but we also significantly improve the prediction accuracy by making the model more consistent with the format of the data (see Tables 1, 2). In addition to testing eSABRE, we have also looked at the best way of selecting random‐effects coefficients. In Section 5.2 we proposed biWAIC as a new method for selecting random‐effect components in the latent variable models with the structure of eSABRE, where it is not possible to apply iWAIC. The results of Table 3 and Figs 4 and 5 show how biWAIC properly accounts for the distribution of the latent variables, resulting in a more realistic number of random‐effect components being included compared with nWAIC and a smaller computational cost than Bayesian ICV.

Section 7 demonstrates how eSABRE, together with biWAIC, can be effectively applied to large real life influenza data sets. In Section 7 we show how the improvement in computational efficiency demonstrated in Table 1, part (b), allows us to make use of the full H1N1 data set rather than a reduced version as was required for the conjugate SABRE model in Davies *et al*. (2017). The results from using the full H1N1 data set and properly accounting for the error in the data collection process through eSABRE show an improvement in the selection of antigenic variables in the H1N1 data sets.

Further work involves investigating how different types of amino acid change at the same residue position on the structure affect antigenic variability. The data currently consist of indicators of amino acid change that are identical regardless of which particular amino acids are involved. However, given the range of biophysical properties among different amino acids, we expect the antigenic effect of a change to depend on both the location on the structure and the particular amino acids that are involved. In Reeve *et al*. (2016) and Harvey *et al*. (2016) variables were used that indicated a particular amino acid change at a given location. This will significantly increase the number of variables that must be selected from and therefore it is likely that the model will require additional information to prevent spurious results. Latent Gaussian processes can be used to include the additional information, e.g. Filippone *et al*. (2013), and will enable us to account for

- differences in the antigenic effect of amino acid substitutions that depend on the amino acids involved and
- similarities between changes of a certain type that occur at the same, or similar, locations on the protein surface.

## Supporting information

‘Improving the identification of antigenic sites in the H1N1 Influenza virus through accounting for the experimental structure in a sparse hierarchical Bayesian model—supplementary materials’.
