# Supplementary material for: Improving the identification of antigenic sites in the H1N1 influenza virus through accounting for the experimental structure in a sparse hierarchical Bayesian model
Source: J R Stat Soc Ser C Appl Stat. 2019 Feb 3;68(4):859–85. doi: 10.1111/rssc.12338 (PMC6774336; doi:10.1111/rssc.12338)
Supplement: Supplementary file 1 — ‘Improving the identification of antigenic sites in the H1N1 Influenza virus through accounting for the experimental structure in a sparse hierarchical Bayesian model—supplementary materials’. [file RSSC-68-859-s001.pdf]

Improving the identification of antigenic sites in the H1N1 Influenza virus through accounting for the experimental structure in a sparse hierarchical Bayesian model - Supplementary Materials

Vinny Davies[1,2,*], William T. Harvey[3], Richard Reeve[3] and Dirk Husmeier[2]

[1]School of Computing Science, University of Glasgow, Glasgow, UK. [2]School of Mathematics and Statistics, University of Glasgow, Glasgow, UK. [3]Boyd Orr Centre for Population and Ecosystem Health, Institute of Biodiversity, Animal Health and Comparative Medicine, College of Medical, Veterinary and Life Sciences, University of Glasgow, Glasgow, UK
[*]Vinny.Davies@Glasgow.ac.uk

This supplementary material provide details which for conciseness were not included in the main paper. More precisely, it gives mathematical derivations of our model and inference equations, as well as additional results for the simulation studies.

1 Conditional Distributions

The conditional distributions for the eSABRE method can be found by using some of the basic results from standard textbooks, e.g. Murphy (2012), where we define $\mathbf{X}^*_{\boldsymbol{\gamma}} = (\mathbf{1}, \mathbf{X}_{\boldsymbol{\gamma}})$, $\mathbf{m}_{\boldsymbol{\gamma}} = (\mu_{w0}, \mu_{w,1}, \ldots, \mu_{w,1}, \mu_{w,2}, \ \ldots, \mu_{w,H})^\top$ and $\boldsymbol{\Sigma}_{\mathbf{w}^*_{\boldsymbol{\gamma}}} = diag(\boldsymbol{\sigma}^2_{\mathbf{w}^*})$ with $\boldsymbol{\sigma}^2_{\mathbf{w}^*} = (\sigma^2_{w0}, \sigma^2_{w,1}, \ldots, \sigma^2_{w,1}, \sigma^2_{w,2}, \ldots, \sigma^2_{w,H})^\top$.

Using standard results for conditional Gaussian distributions and Figure 2 from the main paper, we can calculate the conditional distributions for $\boldsymbol{\mu}_{\mathbf{y}}$, $\mathbf{w}^*_{\boldsymbol{\gamma}}$, $\mathbf{b}$ and μ_w, where we define $\boldsymbol{\theta}$ to be a vector of all the parameters and hyperparameters:

$$p(\boldsymbol{\mu}_{\mathbf{y}}|\boldsymbol{\theta}', \mathbf{X}^*_{\boldsymbol{\gamma}}, \mathbf{Z}, \mathbf{y}) \propto \mathcal{N}(\mathbf{y}|\mathbf{M}\boldsymbol{\mu}_{\mathbf{y}} + \mathbf{Z}\mathbf{b}, \sigma^2_y\mathbf{I})\mathcal{N}(\boldsymbol{\mu}_{\mathbf{y}}|\mathbf{1}w_0 + \mathbf{X}_{\boldsymbol{\gamma}}\mathbf{w}_{\boldsymbol{\gamma}}, \sigma^2_\varepsilon\mathbf{I}) \quad (1)$$

$$\propto \mathcal{N}(\boldsymbol{\mu}_{\mathbf{y}}|\mathbf{V}_{\mathbf{y}}(\mathbf{M}^\top(\mathbf{y} - \mathbf{Z}\mathbf{b})/\sigma^2_y + \mathbf{X}^*_{\boldsymbol{\gamma}}\mathbf{w}^*_{\boldsymbol{\gamma}}/\sigma^2_\varepsilon), \mathbf{V}_{\mathbf{y}}) \quad (2)$$

where $\mathbf{V}_{\mathbf{y}} = (1/\sigma^2_\varepsilon\mathbf{I} + \mathbf{M}^\top\mathbf{M}/\sigma^2_y)^{-1}$.

$$p(\mathbf{w}^*_{\boldsymbol{\gamma}}|\boldsymbol{\theta}', \mathbf{X}^*_{\boldsymbol{\gamma}}, \mathbf{Z}, \mathbf{y}) \propto \mathcal{N}(\boldsymbol{\mu}_{\mathbf{y}}|\mathbf{X}^*_{\boldsymbol{\gamma}}\mathbf{w}^*_{\boldsymbol{\gamma}}, \sigma^2_\varepsilon\mathbf{I})\mathcal{N}(\mathbf{w}^*_{\boldsymbol{\gamma}}|\mathbf{m}_{\boldsymbol{\gamma}}, \sigma^2_\varepsilon\boldsymbol{\Sigma}_{\mathbf{w}^*_{\boldsymbol{\gamma}}}) \quad (3)$$

$$\propto \mathcal{N}(\mathbf{w}^*_{\boldsymbol{\gamma}}|\mathbf{V}_{\mathbf{w}^*_{\boldsymbol{\gamma}}}\mathbf{X}^{*\top}_{\boldsymbol{\gamma}}\boldsymbol{\mu}_{\mathbf{y}} + \mathbf{V}_{\mathbf{w}^*_{\boldsymbol{\gamma}}}\boldsymbol{\Sigma}^{-1}_{\mathbf{w}^*_{\boldsymbol{\gamma}}}\mathbf{m}_{\boldsymbol{\gamma}}, \sigma^2_\varepsilon\mathbf{V}_{\mathbf{w}^*_{\boldsymbol{\gamma}}}) \quad (4)$$

where $\mathbf{V}_{\mathbf{w}^*_{\boldsymbol{\gamma}}} = (\mathbf{X}^{*\top}_{\boldsymbol{\gamma}}\mathbf{X}^*_{\boldsymbol{\gamma}} + \boldsymbol{\Sigma}^{-1}_{\mathbf{w}^*_{\boldsymbol{\gamma}}})^{-1}$.

$$p(\mathbf{b}|\boldsymbol{\theta}', \mathbf{X}^*_{\boldsymbol{\gamma}}, \mathbf{Z}, \mathbf{y}) \propto \mathcal{N}(\mathbf{y}|\mathbf{M}\boldsymbol{\mu}_{\mathbf{y}} + \mathbf{Z}\mathbf{b}, \sigma^2_y\mathbf{I})\mathcal{N}(\mathbf{b}|\mathbf{0}, \boldsymbol{\Sigma}_b) \quad (5)$$

$$\propto \mathcal{N}(\mathbf{b}|\tfrac{1}{\sigma^2_y}\mathbf{V}_{\mathbf{b}}\mathbf{Z}^\top(\mathbf{y} - \mathbf{M}\boldsymbol{\mu}_{\mathbf{y}}), \mathbf{V}_{\mathbf{b}}) \quad (6)$$

where $\mathbf{V}_{\mathbf{b}} = (\frac{1}{\sigma^2_y}\mathbf{Z}^\top\mathbf{Z} + \boldsymbol{\Sigma}^{-1}_{\mathbf{b}})^{-1}$.

$$p(\mu_w|\boldsymbol{\theta}', \mathbf{X}^*_{\boldsymbol{\gamma}}, \mathbf{Z}, \mathbf{y}) \propto \mathcal{N}(\mathbf{w}_{\boldsymbol{\gamma}}|\mathbf{1}\mu_w, \sigma^2_\varepsilon\sigma^2_w\mathbf{I})\mathcal{N}(\mu_w|\mu_0, \sigma^2_0\sigma^2_\varepsilon) \quad (7)$$

$$\propto \mathcal{N}(\mu_w|V_{\mu_w}(\mathbf{1}\mathbf{w}_{\boldsymbol{\gamma}}/\sigma^2_w + \mu_0/\sigma^2_0), \sigma^2_\varepsilon V_{\mu_w}) \quad (8)$$

where $V_{\mu_w} = (1/\sigma_0^2 + ||\mathbf{w}_{\boldsymbol{\gamma}}||/\sigma_w^2)^{-1}$.

We can then calculate the conditional distributions of the variance parameters:

$$p(\sigma_y^2|\boldsymbol{\theta}', \mathbf{X}^*_{\boldsymbol{\gamma}}, \mathbf{Z}, \mathbf{y}) \propto \mathcal{N}(\mathbf{y}|\mathbf{M}\boldsymbol{\mu}_{\mathbf{y}} + \mathbf{Z}\mathbf{b}, \sigma_y^2\mathbf{I})\mathcal{IG}(\sigma_y^2|\alpha_y, \beta_y) \tag{9}$$

$$\propto \mathcal{IG}(\sigma_y^2|\ ||\mathbf{y}||/2 + \alpha_y, \tfrac{1}{2}(\mathbf{y} - \mathbf{M}\boldsymbol{\mu}_{\mathbf{y}} - \mathbf{Z}\mathbf{b})^\top(\mathbf{y} - \mathbf{M}\boldsymbol{\mu}_{\mathbf{y}} - \mathbf{Z}\mathbf{b})) \tag{10}$$

$$p(\sigma_w^2|\boldsymbol{\theta}', \mathbf{X}^*_{\boldsymbol{\gamma}}, \mathbf{Z}, \mathbf{y}) \propto \mathcal{N}(\mathbf{w}_{\boldsymbol{\gamma}}|\mathbf{I}\mu_w, \sigma_\varepsilon^2\sigma_w^2\mathbf{I})\mathcal{IG}(\sigma_w^2|\alpha_w, \beta_w) \tag{11}$$

$$\propto \mathcal{IG}(\sigma_w^2|\ ||\mathbf{w}_{\boldsymbol{\gamma}}||/2 + \alpha_w, \tfrac{1}{2\sigma_\varepsilon^2}(\mathbf{w}_{\boldsymbol{\gamma}} - \mathbf{I}\mu_w)^\top(\mathbf{w}_{\boldsymbol{\gamma}} - \mathbf{I}\mu_w)) \tag{12}$$

$$p(\sigma_{b,g}^2|\boldsymbol{\theta}', \mathbf{X}^*_{\boldsymbol{\gamma}}, \mathbf{Z}, \mathbf{y}) \propto \mathcal{N}(\mathbf{b}_g|\mathbf{0}, \sigma_{b,g}^2\mathbf{I})\mathcal{IG}(\sigma_{b,g}^2|\alpha_{b,g}, \beta_{b,g}) \tag{13}$$

$$\propto \mathcal{IG}(\sigma_{b,g}^2|\ ||\mathbf{b}_g||/2 + \alpha_{b,g}, \beta_{b,g} + \tfrac{1}{2}\mathbf{b}_g^\top\mathbf{b}_g) \tag{14}$$

where we sample for each g separately.

$$p(\sigma_\varepsilon^2|\boldsymbol{\theta}', \mathbf{X}^*_{\boldsymbol{\gamma}}, \mathbf{Z}, \mathbf{y}) \propto \mathcal{N}(\boldsymbol{\mu}_{\mathbf{y}}|\mathbf{X}^*_{\boldsymbol{\gamma}}\mathbf{w}^*_{\boldsymbol{\gamma}}, \sigma_\varepsilon^2\mathbf{I})\mathcal{N}(\mathbf{w}^*_{\boldsymbol{\gamma}}|\mathbf{m}_{\boldsymbol{\gamma}}, \sigma_\varepsilon^2\boldsymbol{\Sigma}_{\mathbf{w}^*_{\boldsymbol{\gamma}}})\mathcal{N}(\mu_{\mathbf{w}}|\mu_0, \sigma_\varepsilon^2\sigma_0^2)\mathcal{IG}(\sigma_\varepsilon^2|\alpha_\varepsilon, \beta_\varepsilon) \tag{15}$$

$$\propto \mathcal{IG}(\sigma_\varepsilon^2|(||\boldsymbol{\mu}_{\mathbf{y}}|| + ||\mathbf{w}^*_{\boldsymbol{\gamma}}|| + 1)/2 + \alpha_\varepsilon, \beta_\varepsilon + \tfrac{1}{2}R_{\sigma_\varepsilon^2}). \tag{16}$$

where we give $R_{\sigma_\varepsilon^2}$ as:

$$\begin{aligned} R_{\sigma_\varepsilon^2} &= (\boldsymbol{\mu}_{\mathbf{y}} - \mathbf{X}^*_{\boldsymbol{\gamma}}\mathbf{w}^*_{\boldsymbol{\gamma}})^\top(\boldsymbol{\mu}_{\mathbf{y}} - \mathbf{X}^*_{\boldsymbol{\gamma}}\mathbf{w}^*_{\boldsymbol{\gamma}}) \\ &\quad + (\mathbf{w}^*_{\boldsymbol{\gamma}} - \mathbf{m}_{\boldsymbol{\gamma}})^\top\boldsymbol{\Sigma}^{-1}_{\mathbf{w}^*_{\boldsymbol{\gamma}}}(\mathbf{w}^*_{\boldsymbol{\gamma}} - \mathbf{m}_{\boldsymbol{\gamma}}) + (\mu_{\mathbf{w}} - \mu_0)^\top(\mu_{\mathbf{w}} - \mu_0)/\sigma_0^2 \end{aligned} \tag{17}$$

Finally we calculate the distribution for π:

$$p(\pi|\boldsymbol{\theta}', \mathbf{X}^*_{\boldsymbol{\gamma}}, \mathbf{Z}, \mathbf{y}) \propto \left\{\prod_{j=1}^{J}\text{Bern}(\gamma_j|\pi)\right\}\mathcal{B}(\pi|\alpha_\pi, \beta_\pi) \tag{18}$$

$$\propto \beta(\pi|\ \alpha_\pi + ||\boldsymbol{\gamma}||, \beta_\pi + J - ||\boldsymbol{\gamma}||). \tag{19}$$

1.1 Sampling $\boldsymbol{\gamma}$

In order to sample $\boldsymbol{\gamma}$ we use collapsing methods as detailed in Section 4 of the main paper. Following the method proposed in Davies et al. (2017) we integrate over μ_w, $\mathbf{w}^*_{\boldsymbol{\gamma}}$, π, and σ_ε^2, however in the case of the eSABRE method are left with a conditional distribution that includes $\boldsymbol{\mu}_{\mathbf{y}}$ but not $\mathbf{y}$, leading to the increased computational efficiency discussed and tested in Sections 4.1 and 8 of the main paper:

$$p(\gamma|\boldsymbol{\theta}', \mathbf{X}^*_{\boldsymbol{\gamma}}, \mathbf{Z}, \mathbf{y}) \propto \int p(\gamma, \pi, \sigma_\varepsilon^2, \mathbf{w}^*_{\boldsymbol{\gamma}}, \mu_w|\boldsymbol{\theta}', \mathbf{X}^*_{\boldsymbol{\gamma}}, \mathbf{Z}, \mathbf{y})d\mu_w d\mathbf{w}^*_{\boldsymbol{\gamma}} d\pi d\sigma_\varepsilon^2 \tag{20}$$

$$\propto \int p(\boldsymbol{\gamma}|\pi)p(\pi)p(\boldsymbol{\mu}_{\mathbf{y}}|\mathbf{w}^*_{\boldsymbol{\gamma}}, \sigma_\varepsilon^2, \mathbf{X}^*_{\boldsymbol{\gamma}})p(\mathbf{w}^*_{\boldsymbol{\gamma}}|\mu_w, \sigma_\varepsilon^2)p(\mu_w)p(\sigma_\varepsilon^2)d\mu_w d\mathbf{w}^*_{\boldsymbol{\gamma}} d\pi d\sigma_\varepsilon^2 \tag{21}$$

$$\begin{aligned} &\propto C_\pi \int \mathcal{N}(\boldsymbol{\mu}_{\mathbf{y}}|\mathbf{X}^*_{\boldsymbol{\gamma}}\mathbf{w}^*_{\boldsymbol{\gamma}}, \sigma_\varepsilon^2\mathbf{I})\mathcal{N}(\mathbf{w}^*_{\boldsymbol{\gamma}}|\mathbf{m}_{\boldsymbol{\gamma}}, \sigma_\varepsilon^2\boldsymbol{\Sigma}_{\mathbf{w}^*_{\boldsymbol{\gamma}}})\mathcal{N}(\mu_w|\mu_0, \sigma_\varepsilon^2\sigma_0^2) \\ &\qquad \mathcal{IG}(\sigma_\varepsilon^2|\alpha_\varepsilon, \beta_\varepsilon)d\mu_w d\mathbf{w}^*_{\boldsymbol{\gamma}} d\sigma_\varepsilon^2 \end{aligned} \tag{22}$$

$$\begin{aligned} &\propto C_\pi \int \mathcal{N}(\boldsymbol{\mu}_{\mathbf{y}}|\mathbf{X}^*_{\boldsymbol{\gamma}}\mathbf{w}^*_{\boldsymbol{\gamma}}, \sigma_\varepsilon^2\mathbf{I})\mathcal{N}(\mathbf{w}^*_{\boldsymbol{\gamma}}|\mathbf{m}_{\boldsymbol{\gamma},\mathbf{0}}, \sigma_\varepsilon^2[\boldsymbol{\Sigma}_{\mathbf{w}^*_{\boldsymbol{\gamma}}} + \mathbf{V}_{\boldsymbol{\gamma},\mathbf{0}}]) \\ &\qquad \mathcal{IG}(\sigma_\varepsilon^2|\alpha_\varepsilon, \beta_\varepsilon)d\mathbf{w}^*_{\boldsymbol{\gamma}} d\sigma_\varepsilon^2 \end{aligned} \tag{23}$$

$$\propto C_\pi \int \mathcal{N}(\boldsymbol{\mu}_{\mathbf{y}}|\mathbf{X}_{\boldsymbol{\gamma}}\mathbf{m}_{\boldsymbol{\gamma},\mathbf{0}}, \sigma_\varepsilon^2[\mathbf{I} + \mathbf{X}_{\boldsymbol{\gamma}}[\boldsymbol{\Sigma}_{\mathbf{w}^*_{\boldsymbol{\gamma}}} + \mathbf{V}_{\boldsymbol{\gamma},\mathbf{0}}]\mathbf{X}^\top_{\boldsymbol{\gamma}}])\mathcal{IG}(\sigma_\varepsilon^2|\alpha_\varepsilon, \beta_\varepsilon)d\sigma_\varepsilon^2 \tag{24}$$

$$\propto C_\pi|\boldsymbol{\Sigma}_{\boldsymbol{\gamma}}|^{-\frac{1}{2}}[\beta_\varepsilon + \tfrac{1}{2}(\boldsymbol{\mu}_{\mathbf{y}} - \mathbf{X}^*_{\boldsymbol{\gamma}}\mathbf{m}_{\boldsymbol{\gamma},\mathbf{0}})^\top\boldsymbol{\Sigma}^{-1}_{\boldsymbol{\gamma}}(\boldsymbol{\mu}_{\mathbf{y}} - \mathbf{X}^*_{\boldsymbol{\gamma}}\mathbf{m}_{\boldsymbol{\gamma},\mathbf{0}})]^{-(N/2+\alpha_\varepsilon)} \tag{25}$$

where $C_\pi = \frac{\Gamma(||\gamma||+\alpha_\pi)\Gamma(J-||\gamma||+\beta_\pi)}{\Gamma(J+\alpha_\pi+\beta_\pi)}$, $\mathbf{\Sigma_\gamma} = [\mathbf{I} + \mathbf{X}^*_\gamma[\mathbf{\Sigma}_{\mathbf{w}^*_\gamma} + \mathbf{V}_{\gamma,\mathbf{0}}]\mathbf{X}^{*\top}_\gamma]$, $\mathbf{m}_{\gamma,\mathbf{0}} = (\mu_{w_0}, \mu_0, \ldots, \mu_0)^\top$ with μ_0 repeated with length $||\mathbf{w}_\gamma||$ dependent on $\boldsymbol{\gamma}$, and $\mathbf{V}_{\gamma,\mathbf{0}}$ is a block diagonal matrix of $(0, \sigma_0^2)$ where the square blocks have length 1 and $||\mathbf{w}_\gamma||$ respectively.

We can use the Woodbury identity and the extended Sylvester's determinant theorem to speed up the computations and give the following conditional posterior distribution:

$$\begin{aligned}
\log\ p(\boldsymbol{\gamma}|\boldsymbol{\theta}', \mathbf{X}^*_\gamma, \mathbf{Z}, \mathbf{y}) &\propto \log\Gamma(||\boldsymbol{\gamma}|| + \alpha_\pi) + \log\Gamma(J - ||\boldsymbol{\gamma}|| + \beta_\pi) \\
&- \log\Gamma(J + \alpha_\pi + \beta_\pi) - \tfrac{1}{2}\log|\mathbf{\Sigma}_{\mathbf{w}^*_\gamma} + \mathbf{V}_{\gamma,\mathbf{0}}| - \tfrac{1}{2}\log|[\mathbf{\Sigma}_{\mathbf{w}^*_\gamma} + \mathbf{V}_{\gamma,\mathbf{0}}]^{-1} + \mathbf{X}^{*\top}_\gamma \mathbf{X}^*_\gamma| \\
&- (\tfrac{N}{2} + \alpha_\varepsilon)\log(\beta_\varepsilon + \tfrac{1}{2}(\boldsymbol{\mu}_\mathbf{y} - \mathbf{X}^*_\gamma \mathbf{m}_{\gamma,\mathbf{0}})^\top \\
&[\mathbf{I} - \mathbf{X}^*_\gamma([\mathbf{\Sigma}_{\mathbf{w}^*_\gamma} + \mathbf{V}_{\gamma,\mathbf{0}}]^{-1} + \mathbf{X}^{*\top}_\gamma \mathbf{X}^*_\gamma)^{-1}\mathbf{X}^{*\top}_\gamma)](\boldsymbol{\mu}_\mathbf{y} - \mathbf{X}^*_\gamma \mathbf{m}_{\gamma,\mathbf{0}})). \qquad (26)
\end{aligned}$$

1.2 Collapsing Within Conditional Distributions

In order to sample the eSABRE method via the collapsing scheme suggested in Section 4 of the main paper, we must derive the collapsed conditional distributions for σ^2_ε and μ_w. The conditional distribution of $\boldsymbol{\gamma}$ is derived in Section 1.1, while (3) and (18) give the distributions for π and $\mathbf{w}^*_\gamma$. The conditional distribution for σ^2_ε can then be derived as follows:

$$p(\sigma^2_\varepsilon|\boldsymbol{\gamma}) \propto \mathcal{N}(\boldsymbol{\mu}_\mathbf{y}|\mathbf{X}^*_\gamma \mathbf{m}_{\gamma,\mathbf{0}}, \sigma^2_\varepsilon \mathbf{\Sigma}_\gamma)\mathcal{IG}(\sigma^2_\varepsilon|\alpha_\varepsilon, \beta_\varepsilon) \qquad (27)$$

$$\propto \mathcal{IG}(\sigma^2_\varepsilon|||\boldsymbol{\mu}_\mathbf{y}||/2 + \alpha_\varepsilon, \beta_\varepsilon + \tfrac{1}{2}(\boldsymbol{\mu}_\mathbf{y} - \mathbf{X}^*_\gamma \mathbf{m}_{\gamma,\mathbf{0}})^\top \mathbf{\Sigma}^{-1}_\gamma(\boldsymbol{\mu}_\mathbf{y} - \mathbf{X}^*_\gamma \mathbf{m}_{\gamma,\mathbf{0}})) \qquad (28)$$

where the first distribution is taken from results in Section 1.1 and the definitions of $\mathbf{m}_{\gamma,\mathbf{0}}$ and $\mathbf{\Sigma}_\gamma$ are given in Section 1.1. Finally we can give the conditional distribution of μ_w as follows:

$$p(\mu_w|\sigma^2_\varepsilon, \boldsymbol{\gamma}) \propto \int \mathcal{N}(\boldsymbol{\mu}_\mathbf{y}|\mathbf{X}^*_\gamma \mathbf{w}^*_\gamma, \sigma^2_\varepsilon \mathbf{I})\mathcal{N}(\mathbf{w}^*_\gamma|\mathbf{m}_\gamma, \sigma^2_\varepsilon \mathbf{\Sigma}_{\mathbf{w}^*_\gamma})\mathcal{N}(\mu_w|\mu_0, \sigma^2_\varepsilon \sigma^2_0)d\mathbf{w}^*_\gamma \qquad (29)$$

$$\propto \mathcal{N}(\boldsymbol{\mu}_\mathbf{y}|\mathbf{1}\mu_{w_0} + \mathbf{X}_\gamma \mathbf{1}\mu_w, \sigma^2_\varepsilon[\mathbf{I} + \mathbf{X}^*_\gamma \mathbf{\Sigma}_{\mathbf{w}^*_\gamma} \mathbf{X}^{*,\top}_\gamma])\mathcal{N}(\mu_w|\mu_0, \sigma^2_0) \qquad (30)$$

$$\propto \mathcal{N}(\mu_w|V_{\mu_w}[\mu_0/\sigma^2_0 + \mathbf{1}^\top \mathbf{X}^\top_\gamma[\mathbf{I} + \mathbf{X}^*_\gamma \mathbf{\Sigma}_{\mathbf{w}^*_\gamma} \mathbf{X}^{*,\top}_\gamma]^{-1}(\boldsymbol{\mu}_\mathbf{y} - \mathbf{1}\mu_{w_0})], \sigma^2_\varepsilon V_{\mu_w}) \qquad (31)$$

where $\mathbf{V}_{\boldsymbol{\mu}_{\gamma,\mathbf{w}}} = [1/\sigma^2_0 + \mathbf{1}^\top \mathbf{X}^\top_\gamma[\mathbf{I} + \mathbf{X}^*_\gamma \mathbf{\Sigma}_{\mathbf{w}^*_\gamma} \mathbf{X}^{*\top}_\gamma]^{-1}\mathbf{X}_\gamma \mathbf{1}]^{-1}$.

2 Additional Data Information

The 570 reference and test virus pairs represent only a subset of the possible pairs that are available for testing. During influenza surveillance, test viruses are characterised through testing with a panel of relatively antigenically similar reference viruses so there is a systematic missingness pattern (see Harvey et al. (2016), Figure 1 for an illustration of this pattern). This

Table 1: **Table of differences between the eSABRE and conjugate SABRE methods in terms of mean squared error (MSE) of out-of-sample predictive performance.** Out-of-sample predictive performance was computed based on 1,000 out-of-sample observations using sampled parameter values for each method. A negative value shows improved performance for the eSABRE method. Additionally in each table are p-values from t-tests to see whether the differences are significantly different from zero.

	**Δ in MSE**			**P-Value**		
**Obs** \ σ^2_y, σ^2_ε	**0.033**	**0.1**	**0.3**	**0.033**	**0.1**	**0.3**
**500**	-0.010	-0.003	-0.035	< 0.001	< 0.001	< 0.001
**1000**	-0.007	-0.002	-0.280	< 0.001	< 0.001	< 0.001

however is not expected to be detrimental as titres between pairs of distant viruses provide no useful information due to there generally being no cross-reaction between such viruses. We analysed the subset of all collected data comprising all viruses used as both test and reference viruses . This constituted the largest dataset that allowed us to disambiguate between avidity and antigenic effects.

3 Model Selection Strategies

We have used two model selection strategies. For the feature selection, which is of primary interest for the identification of antigenic sites, we have used a Bayesian approach based on a spike-and-slab prior. For the selection of random effect components, on the other hand, we have used an approach based on information criteria, more specifically the widely applicable information criterion (WAIC). While the latter choice was mainly motivated for computational viability, as discussed at the beginning of Section 5 of the main paper, a referee has suggested that we discuss the implications in the context of Lindley's paradox (Lindley, 1957). In a nutshell, Lindley's paradox states that Bayesian model selection increasingly favours the less complex model as the vagueness of the prior relative to the posterior increases, while methods based on hold-out predictive performance (to which WAIC is asymptotically equivalent) are unaffected by the vagueness of the prior. Deeper methodological insight into the nature of this paradox can be obtained from Robert (2014), and strategies for calibration of Bayesian variable selection with out-of-sample cross validation have been discussed in Vehtari and Ojanen (2012) and references therein. We here refer to a recent comprehensive evaluation study, Aderhold et al. (2016), where Bayesian variable selection was systematically compared with WAIC for variations of the prior uncertainty over several orders of magnitude. The upshot is that the effect of Lindley's paradox only manifests itself for extreme degrees of prior uncertainty, and that WAIC and Bayesian model selection tend to give consistent results for prior parameter uncertainties that are realistic in practice. The former finding tallies with the conclusion in Robert (2014) that excessively vague and improper priors should be avoided, as we did in our study. The latter finding provides a practical justification for our approach of combining both model selection approaches.

4 Additional Results

This section contains additonal results for the simulation studies, which for brevity were not included in the main paper.

4.1 Additional results for the influenza inspired simulated datasets

To compare the out-of-sample performance of the eSABRE and conjugate SABRE methods, we used the 2,000 observations from each of the influenza inspired simulated datasets. We compare out-of-sample performance for the models trained on 500 and 1,000 observations respectively, using the remaining 1,000 samples for testing. Based on the generated parameter samples for each of these methods we made predictions for the 1,000 out-of-sample points using the (thinned) parameter samples.

Table 1 shows the difference in mean squared error (MSE) between the eSABRE and conjugate SABRE methods, where a negative value means that there is a lower MSE for the eSABRE method. The results of Table 1 show that the eSABRE method has a lower MSE in each case, with the corresponding p-values showing that this difference is significantly different from zero. This result, along with that of Table 1 of the main paper, show that the eSABRE method outperforms the conjugate SABRE method in terms of both out-of-sample performance and variable selection in all scenarios tested here.

Table 2: **Table of differences in performance between biWAIC and nWAIC for mean out-of-sample (OOS) log-likelihood and mean number of variables included in each model.** The tables give the difference between the (a) mean out-of-sample log-likelihood and (b) mean number of variables included in the model when the random effects factors are selected by biWAIC and nWAIC respectively. A positive value shows (a) a higher mean OOS log-likelihood and (b) a larger number of variables included in the model for when the random effects factors are selected by biWAIC. Additionally in each table are p-values from t-tests to see whether the differences are significantly different from zero.

	**Δ in Mean OOS Log-Likelihood**			**P-Value**		
**P** \ σ^2_ε	**0.1**	**0.3**	**0.5**	**0.1**	**0.3**	**0.5**
**55**	25.21	-67.95	-139.06	0.093	0.204	0.116
**465**	0.22	-1.29	0.12	0.552	0.400	0.612
**1035**	-10.14	-41.38	-30.09	0.598	0.127	0.254

(a) Difference in mean out-of-sample (OOS) log-likelihood

	**Δ in Mean Number of Variables**			**P-Value**		
**P** \ σ^2_ε	**0.1**	**0.3**	**0.5**	**0.1**	**0.3**	**0.5**
**55**	1.96	0.92	1.16	0.046	0.061	0.259
**465**	-0.10	-0.06	-0.04	0.078	0.438	0.676
**1035**	-0.02	-0.15	0.07	0.658	0.372	0.624

(b) Difference in mean number of variables selected in the models

4.2 Additional results for the selection of random effect components

To further compare the performance in selecting random effect components, we have looked at the out-of-sample performance of the selected model when using biWAIC and nWAIC. These results are given in Table 2a, where a positive value shows a higher mean out-of-sample log-likelihood when the random effect factors are selected by biWAIC. Additionally, we have looked at the number of variables (fixed effects) selected in the model, to see whether this is affected by the choice of information criterion. These results are shown in Table 2b, where a positive values indicates that the models selected by biWAIC have more variables in them than the models selected by nWAIC.

The results in Table 2a show that there is no real difference in the out-of-sample log-likelihoods for the models selected by biWAIC and nWAIC respectively. The results show 3 scenarios where the models selected by biWAIC have higher mean out-of-sample log-likelihoods, and 6 where it is higher for nWAIC. In each case, the results were not significant at the 5% significance level.

Similarly, the results of Table 2b show no real difference in the number of variables (fixed effects) selected when biWAIC and nWAIC are used to select the models respectively. There were 4 scenarios where biWAIC had a larger mean number of variables and 5 where it was larger for nWAIC. The only case where we see a p-value less than 0.05 is where $P = 55$ and $\sigma^2_\varepsilon = 0.1$. However after the Bonferroni correction for multiple testing, this result is no longer significant.

4.3 Additional results for the H1N1 data

Figure 1 shows the observed log HI titre values against the mean predicted log HI titre values. Additionally Figure 2 shows examples of typical densities of the predictions of 4 observations. In both cases, the mean predicted log HI titre values are calculated based on the mean of

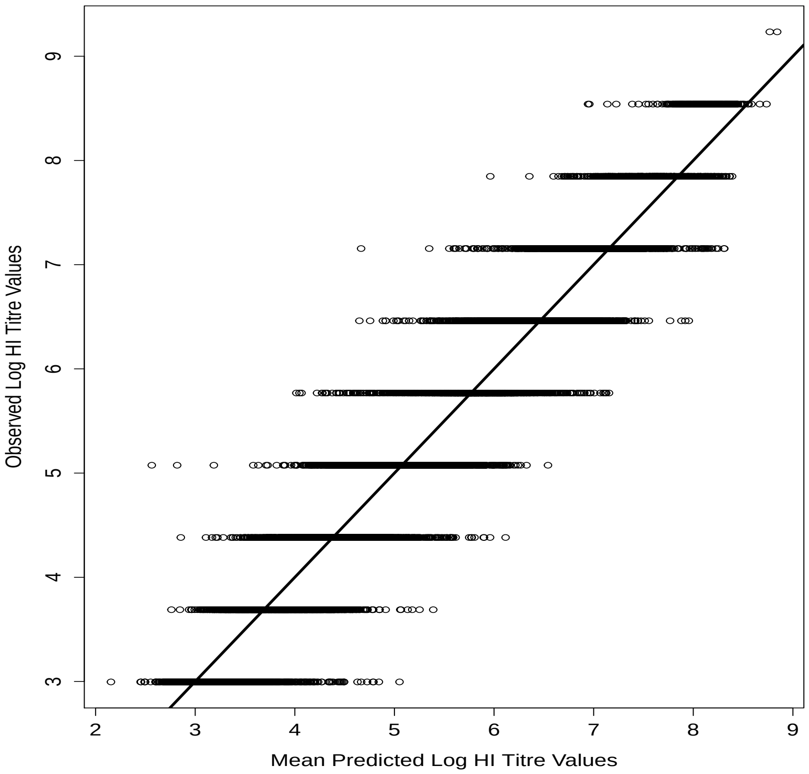

Figure 1: **Plot of the observed log HI titre values against the mean predicted log HI titre values.** Mean predicted log HI titre values are calculated based on the posterior mean of the predictions made by thinned MCMC parameter samples from final model selected by biWAIC.

the prediction made by thinned parameter samples from final model selected by biWAIC. The results in Figure 1 give a good fit between the predicted and true values, as shown by the values being spread around the diagonal line indicating the same predicted and observed values. The somewhat unusual structure to the observed log HI titre values come from the experimental procedure. Virus pairs are only tested at certain dilution ratios, meaning there are only a certain number of log HI titre values possible to observe. These are likely to be noisy values and it is for this reason that our model fits to a continuous value, with the actually true values likely to be between the different possible observed HI titre values.

References

Aderhold, A., D. Husmeier, and M. Grzegorczyk (2016). Approximate Bayesian inference in semi-mechanistic models. *Statistics and Computing 27*(4), 1003–1040.

Davies, V., R. Reeve, W. T. Harvey, F. F. Maree, and D. Husmeier (2017). A sparse hierarchical Bayesian model for detecting relevant antigenic sites in virus evolution. *Computational Statistics 32*(3), 803–843.

Harvey, W. T., D. J. Benton, V. Gregory, J. P. J. Hall, R. S. Daniels, T. Bedford, D. T. Haydon, A. J. Hay, J. W. McCauley, and R. Reeve (2016). Identification of low- and high-impact hemagglutinin amino acid substitutions that drive antigenic drift of influenza A(H1N1) viruses. *PLoS Pathog 12*(4), 1–23.

Lindley, D. V. (1957). A statistical paradox. *Biometrika 44*(1/2), 187–192.

Murphy, K. P. (2012). *Machine learning: a probabilistic perspective.* Cambridge, MA: MIT Press.

Robert, C. P. (2014). On the Jeffreys-Lindley paradox. *Philosophy of Science 81*(2), 216–232.

Vehtari, A. and J. Ojanen (2012). A survey of Bayesian predictive methods for model assessment, selection and comparison. *Statistics Surveys 6*, 142–228.

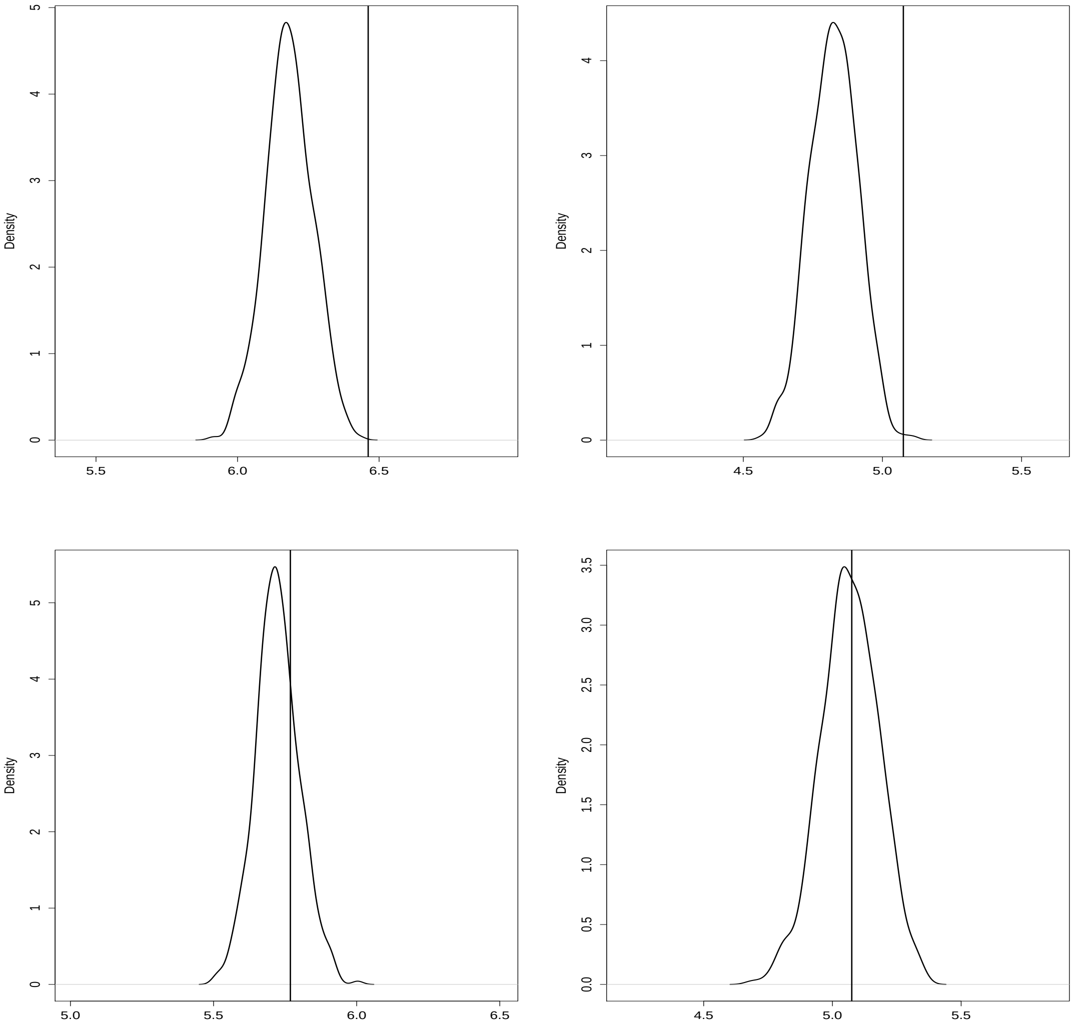

Figure 2: **Plots showing posterior densities of the predictions of 4 randomly selected observations.** Estimates are created by using thinned parameter samples from the eSABRE model selected by biWAIC and then using a kernel density estimator. The true values are plotted as vertical lines.
